# Viscid silk in spider orb webs adheres strongly across surfaces with different roughnesses and surface energies

**DOI:** 10.1242/bio.061802

**Published:** 2025-05-06

**Authors:** Angela M. Alicea-Serrano, K. Zin Htut, Alix J. Coonfield, Katherine Karkosiak, Ali Dhinojwala, Todd A. Blackledge

**Affiliations:** ^1^ School of Polymer Science and Polymer Engineering, The University of Akron, Akron, OH, 44325, USA; ^2^ Department of Biological Sciences, University of Massachusetts Lowell, MA, 01854, USA; ^3^ Division of Biology, Chemistry, and Material Science, Office of Science and Engineering Laboratories, Center for Devices and Radiological Health, Food and Drug Administration, MD, 20993, USA; ^4^ Department of Biology and Integrated Bioscience, The University of Akron, Akron, OH, 44325, USA

**Keywords:** Viscid silk, Adhesion, Orb web, Spider, Aggregate glue, Surface energy

## Abstract

Orb spiders use glue-coated viscid silk in their webs that maximizes adhesive forces by optimizing spreading across insect surfaces while maintaining strong bulk cohesion. While glue adhesion on smooth hydrophilic glass is well understood, insect cuticles vary in wettability and wax coatings that resist glue spreading, potentially allowing insects to escape webs. Here, we tested the adhesiveness of viscid silk on the superhydrophobic lotus leaf, an extreme case of a hydrophobic surface, to explore whether hydrophobic cuticles can help insects evade webs. We compared adhesion of viscid silk on three substrates: natural lotus leaves (superhydrophobic due to waxes and microtopography), lotus leaves treated with oxygen plasma (hydrophilic but maintaining microtopography), and smooth hydrophilic glass. We found that viscid silk adheres better to the superhydrophobic lotus leaves than to other surfaces, but that adhesion was always higher on the lotus leaves, regardless of surface energy. These findings demonstrate that viscid silk is resilient to a wide range of surface hydrophobicity and leverages microtopography to increase adhesion, both of which are vital for generalist predators like orb-weaving spiders and may inspire the development of tunable adhesives with multifunctional applications in biomedical, industrial, and robotic fields.

## Teaser

**Summary:** Spider viscid silk adheres effectively to diverse surfaces, including superhydrophobic lotus leaves, demonstrating its resilience to surface variation.

## INTRODUCTION

Araneidae orb-weaving spiders are among the most successful predators of flying insects due in part to their use of an aqueous glue in the capture spirals of their webs (Blackledge et al., 2009; Bond and Opell, 1998; Coddington and Levi, 1991; Opell and Bond, 2001; Tarakanova and Buehler, 2012). The capture spirals of orb webs are spun from viscid silk and function to retain prey that hit the webs until the spiders subdue them (e.g. Blackledge and Zevenbergen, 2006). Viscid silk is made of an axial fiber of flagelliform silk coated by evenly spaced glue droplets of adhesive aggregate glue, both of which stretch and deform during pull-off from prey by implementing a suspension bridge mechanism that recruits multiple glue droplets to simultaneously transfer forces from droplet to droplet and from droplet to axial fiber (Liao et al., 2015; Opell and Hendricks, 2007). The aggregate glue in viscid silk is made of glycoproteins and hygroscopic low molecular mass compounds (LMMC) (Choresh et al., 2009; Jain et al., 2018; Townley and Tillinghast, 2013; Townley et al., 1991, 2006; Vollrath and Edmonds, 1989; Vollrath and Tillinghast, 1991; Vollrath et al., 1990). While the glycoproteins act as the primary glue agent (Vollrath and Tillinghast, 1991), the LMMCs include salts and lipids that help to keep droplet elastic properties by sequestering atmospheric water, solvating the glycoproteins to facilitate flow (Sahni et al., 2014), and possibly increasing wettability of prey epicuticles (Salles et al., 2006). Upon contact with a substrate, the aggregate glue droplets quickly spread with adhesion being the product of both glue cohesion and its interaction with the surface (Amarpuri et al., 2015) – dependent on the viscosity of the glue and the wettability of the surface. While the mechanism of viscid silk adhesion is very well understood on smooth hydrophilic substrates, particularly glass, viscid silk behavior on more realistic rough and hydrophobic substrates has not been investigated in detail (with a few exceptions; Alicea-Serrano et al., 2021; Diaz et al., 2020; Eisner et al., 1964; Opell and Schwend, 2007).

Insects have evolved a variety of counter strategies that reduce capture by orb spiders. These counter strategies include escape behaviors, sacrificial scales of moths and butterflies, and potentially the microstructure and chemistry of the chitin exoskeleton itself (Blackledge et al., 2009; Penney, 2004). Insect cuticles are multilayered composites of chitin and proteins, and many cuticles are hydrophobic due to specialized protuberant structures or waxes present in the epicuticle (e.g. Watson et al., 2011). Cuticular waxes play a key role in preventing desiccation (Bott et al., 2017) and are also hypothesized to function in several other ways, such as facilitating mating success (Chung and Carroll, 2015). Wettability of a surface, described by the water contact angle, varies based upon the surface free energy dictated by chemistry, and upon the topography or surface roughness (Peethan et al., 2023; Wenzel, 1936). Superhydrophobicity in insect cuticles may function as anti-fouling, anti-fogging, or anti-reflectance surfaces (e.g. Peethan, 2023). Anti-wetting is also hypothesized to function as protection against spider webs by preventing effective spreading of adhesive glue droplets (Masters and Eisner, 1990; Watson et al., 2011). Some moth-specialist spiders in the subfamily Cyrtarachninae use large, over-lubricated glue droplets for quick spreading underneath superhydrophobic cuticles (Cartan and Miyashita, 2000; Diaz et al., 2018, 2020) and cribellate spiders sometimes leverage capillary forces of epicuticular waxes of insects along the adhesive nanofibers to enhance adhesion (Bott et al., 2017). Most ecribellate orb weaving spiders, however, are prey generalists whose water-based glue is maximally adhesive when balancing the contributions of interfacial spreading and bulk cohesion during pull-off (Amarpuri et al., 2015). Thus, poor wetting of superhydrophobic cuticles could provide a very effective defense strategy that has been overlooked by most research on viscid silk function, which investigates adhesion primarily on smooth, hydrophilic glass surfaces.

**Fig. 1.**
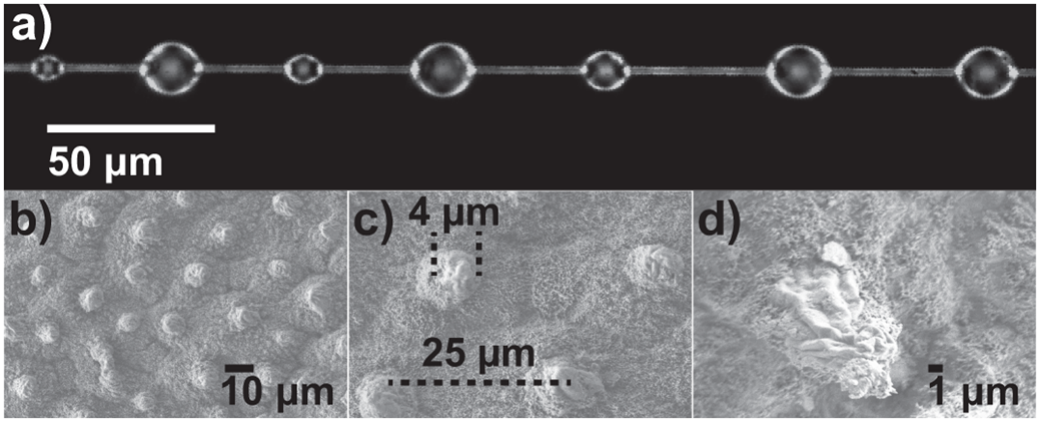
**(A) Viscid silk from orb webs made by *Larinioides cornutus*.** Threads are made of an underlying stretchy flagelliform silk fiber that is covered by evenly spaced glue droplets with 100-300 µm between droplets that range in size from 30-40×20-30 µm, when measured at 50 to 60% RH. (B-D) Lotus leaf is a classic example of a superhydrophobic surface, resulting from its hierarchical structure of micro papillae and nano wax tubules on their tips (not visible). Tips of the papillae are 4-5 µm in diameter, the average distance between the papillae peaks is 15-25 µm, and the papillae are 5-9 µm in height. (C) A higher-magnification view of B, showing finer details of the papillae structure. (D) A higher-magnification view of C, revealing more intricate surface features.

Insect cuticles vary widely in wettability driven by differences in surface free energy and surface roughness – from cockroaches with a water contact angle below 90° (hydrophilic) to mayflies, lacewing flies, butterflies, and moths with water contact angles well above 130° (hydrophobic) (Wagner et al., 1996). Much of this variation in hydrophobicity is confounded with macroscale features such as setae and sacrificial scales that can strongly influence adhesion; our interest was to test the potential for extreme hydrophobicity to function as a defense against viscid silk. We therefore chose to explore the effects of hydrophobicity on silk adhesion using a simpler system with a well-studied topology and surface chemistry – the lotus leaf. The lotus leaf lacks any macroscale setae and, more importantly, ranks as one of the most superhydrophobic surfaces in nature with a water contact angle of around 150°, giving it self-cleaning properties that are of great interest for biomimicry (Martina et al., 2012). Lotus leaf superhydrophobicity results from a hierarchical structure of randomly oriented hydrophobic wax nano-tubules on the top of convex cellular micro-papillae (Fig. 1) (Barthlott and Neinhuis, 1997). While lotus leaves do not resemble typical surfaces of prey for orb-weaving spiders, the surfaces of these leaves are relatively simple since they lack hairs and other potentially entangling macro and hetero-structures, making them a good comparison to the conventional glass surfaces used in various studies on spider silk adhesion. Here, we tested the effects of substrate superhydrophobicity on the adhesion of spider viscid silk, to test the hypothesis that superhydrophobicity can function as an anti-adhesion mechanism to spider webs. Our results show that viscid silk from spider orb webs can adhere to surfaces that vary enormously in surface energy. This should reduce the potential for insects to evolve changes in surface energies of cuticles as anti-adhesion adaptations for spider webs and suggests that a broad range of insects should be ‘available’ as prey to spiders. The underlying mechanisms for this robust adhesion could be exploited in the advancement of technology to create biomimetic adhesives that can stick to a wide range of surfaces.

## RESULTS

### Surface energy

**Fig. 2.**
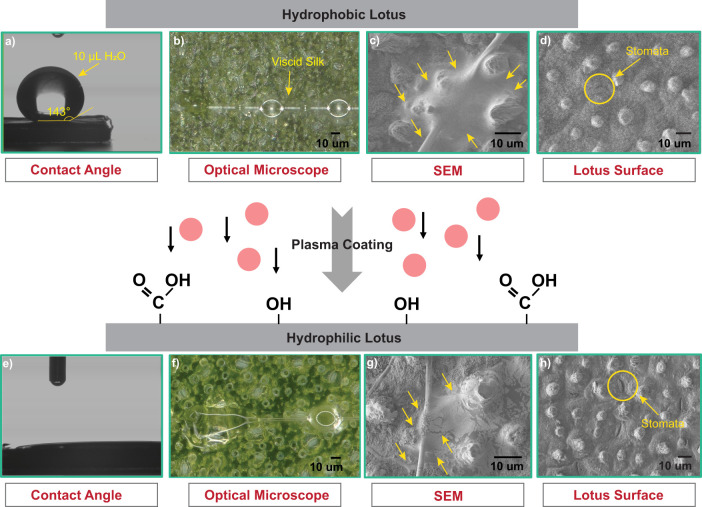
**Diagram showing the process of oxygen plasma-treatment to change the chemistry (surface energy) of the lotus leaf.** Plasma-treatment results in oxidation of non-polar methyl and methylene groups of waxes to polar carboxylic and hydroxyl groups using oxygen ions (represented by red circles) available in the air plasma, changing the surface molecules from a non-wetting to a completely wetting state. Plasma-treatment dramatically increases the ability of water to wet the leaf surface. (A,E) Water contact angle measurements changed from 143.6±1.2° to 0° after plasma treatment. (B,F) Visible enhancement of glue spreading area on the plasma treated hydrophilic lotus seen under an optical microscope (Keyence© VHX-7000 digital microscope). (C,G) SEM shows a thinner adhesive layer near the thread on hydrophilic lotus compared to hydrophobic lotus (indicated with yellow arrows). Note that aggregate glue spread across several papillae in both hydrophobic and hydrophilic lotus. (D,H) Stomata become more visible after the plasma treatment.

Both hydrophilic substrates (hydrophilic lotus and glass) showed contact angles of zero degrees (°), while hydrophobic lotus leaf had an average contact angle of 143.6±1.2° (Fig. 2). Visually, the stomata (pores found in leaves for the exchange of gas and water) in hydrophilic lotus leaves were more noticeable under scanning electron microscopy (SEM) images and were smaller on hydrophobic lotus (Fig. 2). The plasma treatment could etch at low scale roughness on the epidermis layer of lotus (Cortese et al., 2008) and the better visibility of stomata might result from the treatment. No other visual differences between the two lotus treatments were observed. Therefore, we defined our three substrates as smooth/hydrophilic (glass), rough/hydrophilic (plasma treated lotus) and rough/hydrophobic (raw lotus).

### Adhesion

**Fig. 3.**
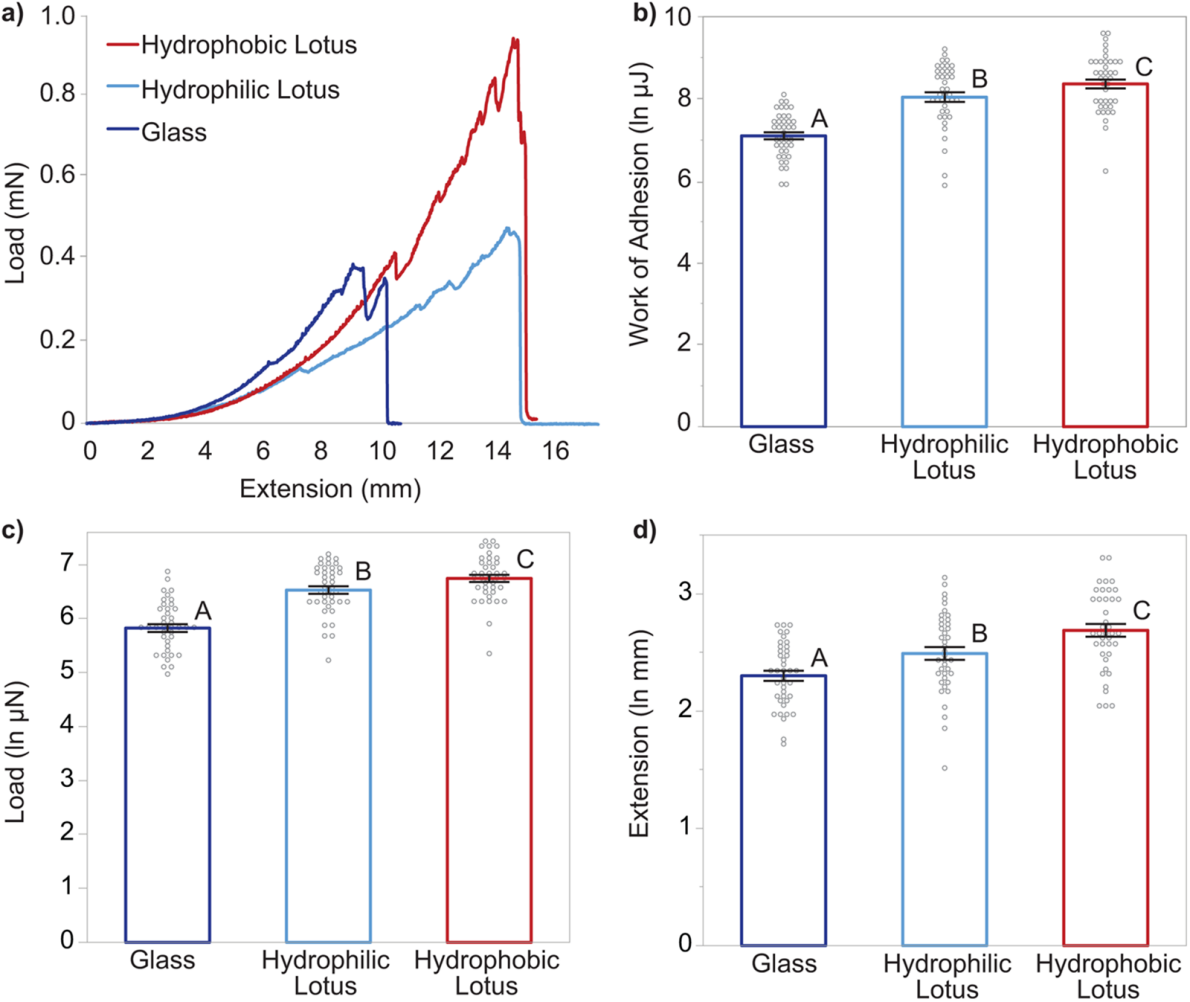
**Pull-off testing results of viscid silk against three substrates: glass (dark blue), hydrophilic lotus (light blue), and hydrophobic lotus (red).** (A) Representative load-extension curves of viscid silk pull-off from each substrate. (B) Work of adhesion is the energy required to pull the viscid silk from the substrate. Glass had the lowest adhesion while the hydrophobic lotus had the highest adhesion [*F*(2, 77.6)=57.90, *P*<0.0001]. Bars represent the mean and error, while grey dots show individual data points. Different letters show statistically significant pair-wise differences determined by Tukey's *post hoc* tests. Work of adhesion is driven by both (C) force of pull-off and (D) extension of the viscid silk, and both showed the same trend as that observed in the work of adhesion [*F*(2, 77.8)=65.89, *P*<0.0001; *F*(2, 77.3)=30.47, *P*<0.0001, respectively]. Threads from 40 webs were tested per substrate (*n*=40).

We tested the adhesive properties of viscid silk from *Larinioides cornutus* webs on raw (rough/hydrophobic) and plasma-treated (rough/hydrophilic) lotus leaves, and glass (smooth/hydrophilic) substrates, which have varying roughness and surface chemistry, and found that the adhesion properties differed between the treatments [*F*(4, 232)=19.69, *P*<0.0001; Wilks' L=0.15, Fig. 3]. We found that viscid silk stuck better to the hydrophobic lotus than the other two substrates and adhesion was lowest on glass compared to lotus leaf, regardless of surface energy. Adhesion was higher on hydrophobic lotus leaf with a mean work±s.e. of 5.25±0.53 μJ, 1.5 times higher than the adhesion of viscid silk on hydrophilic lotus (3.82±0.35 μJ) and ∼4 times higher than the work of glue pulling-off from glass (1.38±0.11 μJ) [*F* (2, 77.6)=57.90, *P*<0.0001; Fig. 3B]. The differences in the work of adhesion for the three substrates were driven by both the force during pull-off and the extension of viscid silk. Hydrophobic lotus had both a significantly higher pull-off force and a higher extension (918±56.2 μN; 15.5±0.8 mm), followed by hydrophilic lotus (745±45.1 μN; 12.8±0.7 mm) and glass (375±28.8 μN; 10.3±0.4 mm) [*F*(2, 77.8)=65.89, *P*<0.0001; *F*(2, 77.3)=30.47, *P*<0.0001; Fig. 3C,D].

### Imaging

**Fig. 4.**
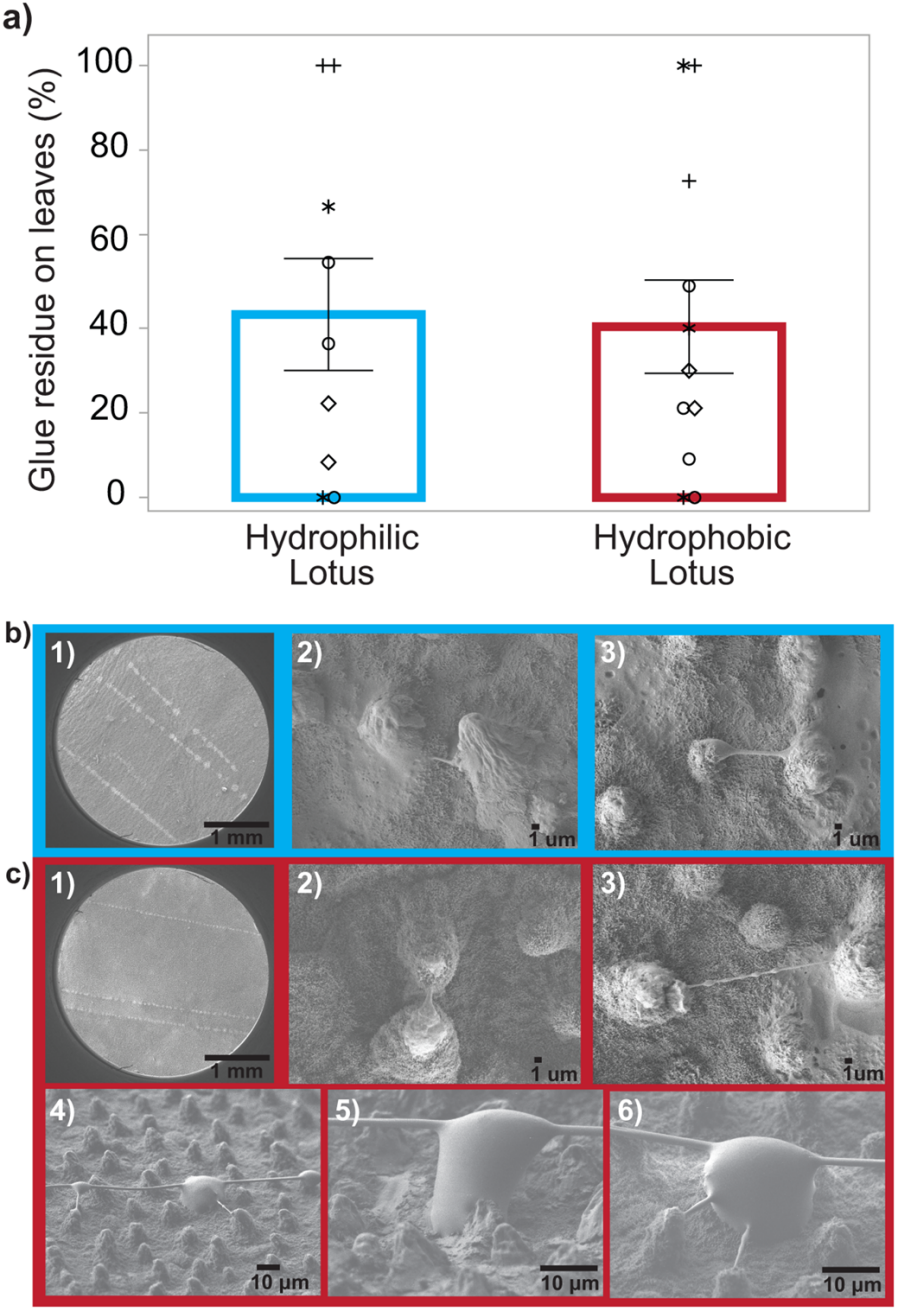
**(A) Percentage of tests with aggregate glue residue was the same for hydrophilic and hydrophobic lotus leaves.** Nine to 14 capture silk threads were tested per leaf sample. The percentage of trials where aggregate glue residue remained on the sample was calculated by dividing the number of trials where aggregate glue failed cohesively by the total number of threads tested in that leaf sample (*n*=9 for hydrophilic lotus, *n*=11 for hydrophobic lotus). Bars represent mean±s.e. for the averages of the samples. Identical symbols represent the same leaf. Multiple papillae are covered by a single droplet of the viscid silk increasing the surface area of contact. (B) Tendrils were rarely observed on hydrophilic lotus and only between closely spaced papillae, causing the tendrils to be short (blue). (C) Tendril formation was almost exclusively found in hydrophobic lotus (red).

After substrates were used to test adhesion, we examined them to see if aggregate glue residue was left behind. Glue left behind could indicate if overspreading of droplets occurred during testing (Amarpuri et al., 2017). We found that about 40% of viscid silks tested on both hydrophobic and hydrophilic lotus left visible aggregate glue residue behind (Fig. 4A). An interesting behavior was observed for the viscid silk glue as it interacted with the lotus rough surface, where glue tendrils formed at the peaks of the papillae (Fig. 4B,C). This behavior was almost exclusively found on the residual glue on hydrophobic lotus leaves, while it was only observed in a few instances for hydrophilic lotus. Furthermore, tendrils observed on hydrophilic lotus only occurred in places where papillae were close together, causing these tendrils to be very short and thick relative to the ones found on hydrophobic lotus.

**Fig. 5.**
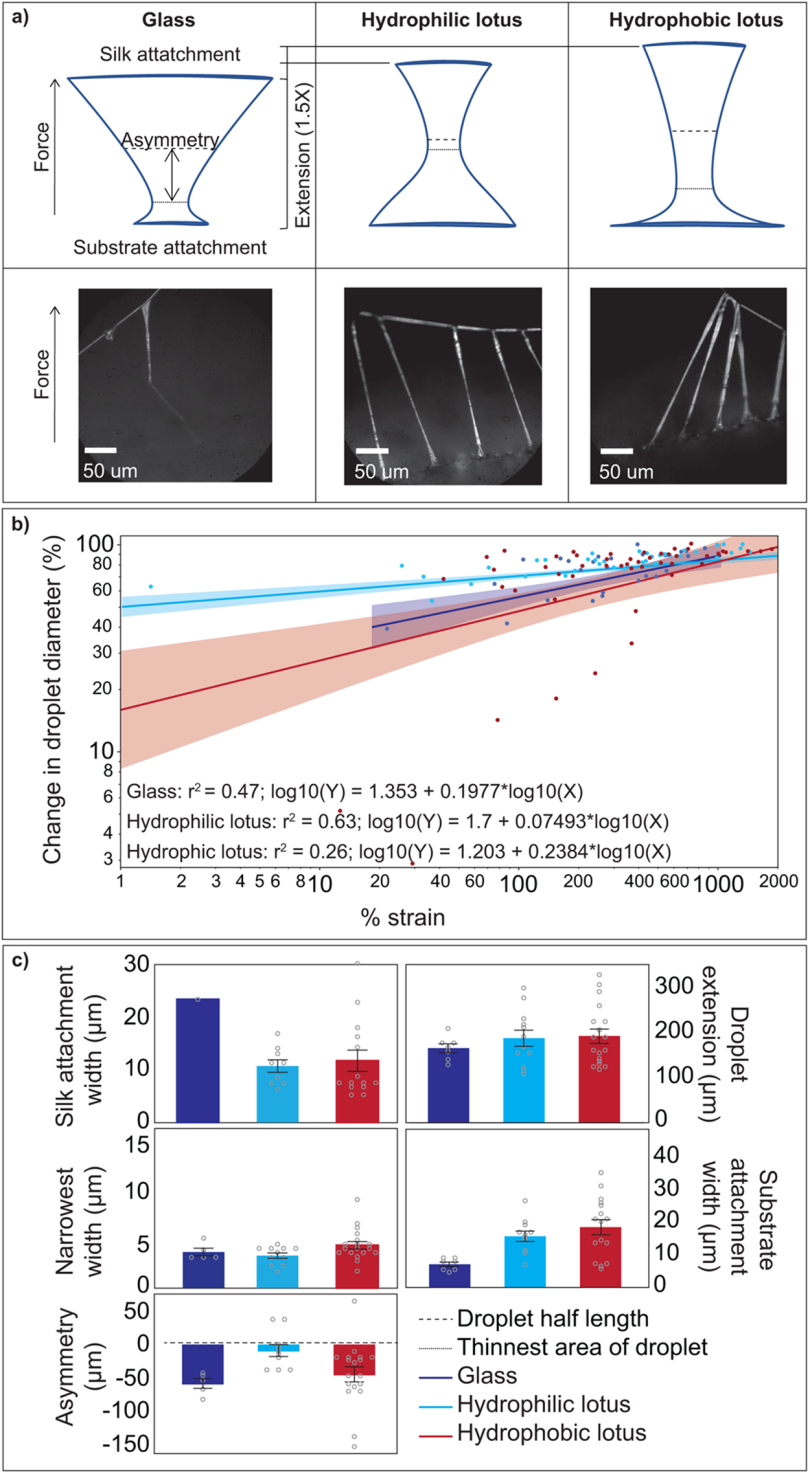
**Glue droplet deformation during pull off.** (A) Diagram of droplet shape (top) and snapshot (bottom) close to failure. Necking occurred on all three substrates but differed in size and location among the three substrates. Diameter of neck is indicated by the solid line while the dotted line indicates the middle of the droplet. Snapshots of the last frames before total pull-off (close to failure) are also shown in Fig. S1 for glass (Fig. S1II), superhydrophobic lotus (Fig. S1VI), and hydrophilic lotus (Fig. S1IV). (B) Linear regression of the percent change in droplet diameter measured at the neck normalized to the original diameter of the suspended droplet, as a function of strain (current length of droplet relative to original diameter of the suspended droplet) for glass (dark blue), hydrophilic lotus (light blue) and hydrophobic lotus (red). While glue droplets on the lotus substrate could reach extensions 1.5X higher than glass, droplets in hydrophilic lotus thins 3X faster than glass and hydrophobic lotus. (C) Droplet measurements at strains close to failure (past 589% strain) for glass (dark blue), hydrophilic lotus (light blue) and hydrophobic lotus (red). We measured the widths at the glue-silk attachment, the glue-substrate attachment, and the narrowest part of the extended droplet. We also measured extension or droplet length to calculate strains, and the asymmetry of the droplet, which refers to the position of the narrowest part of the droplet in respect to the droplet's true center. Bars represent the mean of all droplets measured in frame from two to three strands. Error bars represent the standard error. This graph is meant to be a visual representation of the measurements, and no statistical tests were performed because of the small sample size.

Imaging of glue during pull-off revealed that droplet geometry at strains close to detachment from substrate was different between each substrate (Fig. 5). Droplets that adhered to glass showed an upside down mushroom pillar configuration, with a wider glue attachment to the silk than to the substrate, and an asymmetrical geometry where the thinnest part of the pillar was closer to the glass contact suggesting interfacial failure (Fig. 5). Droplets that adhered to hydrophilic and hydrophobic lotus displayed a geometry like an hourglass, but hydrophilic lotus had a nearly symmetric shape while hydrophobic lotus showed an asymmetrical pillar narrower at the contact base (Fig. 5). The wider contact attachment of the glue on both lotus substrates together with the previously observed results of cohesive failure suggest failure in the bulk. Like adhesion results, extension of the droplets was lower for glass relative to both lotus substrates. While glue droplets detaching from lotus achieve similar strains, the hydrophobic lotus at the same strains have droplets that are wider at the thinnest part, suggesting more material is resisting deformation.

## DISCUSSION

In this study we found that viscid silk sticks remarkably well to the superhydrophobic lotus leaf. The ability of viscid silk to adhere so effectively to one of the most superhydrophobic surfaces in nature suggests that insects have limited potential to evolve hydrophobicity of their cuticles as defenses against spider webs. Our results also reveal the robust adhesion of viscid spider silk across a broad range of surface wettability.

Variation in cuticular chemistry and microstructure plays a key role in how insects interact with their environment (Gullan and Cranston, 2014). For instance, specific cuticular microstructures can help insects thrive in aquatic, aerial, and arid environments (e.g. plastrons help with oxygen retention in aquatic environments; bristles, scales and microtrichia can decrease friction between air and cuticle to enhance aerodynamics; and leaf-like bristles aid in water retention in arid conditions) (Gorb, 2001). Some cuticular features may have evolved as anti-predator specializations against ubiquitous spider webs in the environment. For example, the detachable scales in moth and butterfly wings, and detachable hairs in caddisflies and lacewings allow these insects to rapidly escape most orb webs by sacrificing or shedding these layers (Eisner et al., 1964; Masters and Eisner, 1990; Nentwig, 1982). Long and widely spaced setae on the abdomens of flies prevent aggregate droplets on viscid silk from contacting exposed cuticle, lowering adhesion (Opell and Schwend, 2007). Spiders even protect themselves from sticking to their own webs via a protective surface layer of lipids on their tarsal setae causing lower adhesion than when the layer is removed with solvents (Briceño and Eberhard, 2012), suggesting insects could potentially use a chemical layer as protection too. However, our results argue that cuticular hydrophobicity on its own is unlikely to evolve as a general defense against orb webs because the viscid silk adheres well across a broad range in wettability.

We found that viscid glue adheres better to superhydrophobic lotus leaf than to smooth glass. We suggest that microscale differences in surface roughness were at least as important as chemistry (surface energy) for explaining the 392% higher adhesion of viscid silk on lotus leaf than to glass because viscid silk also adhered 240% better to plasma treated hydrophilic lotus leaf compared to glass. Roughness likely improves adhesion in two ways, increasing the total effective surface area for glue to adhere to within a given space and viscous dissipation between the glue and papillae, a process where the work done by a fluid on surfaces is transformed into heat. Surface topology is known to modulate adhesion for scales larger than 1 µm (Fuller and Tabor, 1975). For soft adhesives, adhesion can be enhanced on rough surfaces because the increased contact area allows for an increase in the effective interfacial energy (e.g. Gullan and Cranston, 2014). Macroscale cuticular features of insects, such as hairs and setae, improved adhesion of viscid silk by increasing the total effective surface area of adhesion when setae were smaller than glue droplets but reduced adhesion when setae were much larger than glue droplets and therefore prevented effective contact (Opell and Schwend, 2007). Viscid silk used in our study, from webs of *Larinioides cornutus*, have droplets that are approximately 20 times larger than lotus papillae (the tip of the papillae ranges from 4-5 µm and the distance between each papillae peak are 15-25 µm as in Fig. 1). Many of these papillae can be covered by a single droplet, which have a circumference of around 128 µm in suspended form (measured at 65% RH; Fig. 2, optical microscope, and SEM). Therefore, viscid silk from *L. cornutus* can effectively contact the lotus substrates optimizing adhesion. However, future investigation should explore the interaction between microscale surface roughness of insect cuticles and spider silk adhesion as there is great variation not only in insect cuticles but also in the size and material properties of aggregate glues among other generalist orb weavers that could yield different results.

While adhesion was much greater on both lotus surfaces compared to glass, natural hydrophobic lotus surfaces also showed a 152% increase in adhesion compared to the plasma-treated hydrophilic lotus. The higher adhesion to hydrophobic lotus can be explained by differences in the spreading behavior (Amarpuri et al., 2015, 2017). There is an ‘ideal’ mushroom pillar shape that optimizes adhesion during pull-off of glue droplets by distributing stress equally at the glue–substrate interface (Amarpuri et al., 2017). We think that on both lotus substrates the outer edge of the glue droplet gets pinned in place, but less work is generated by the hydrophilic lotus, because a thinner adhesive layer of glue (smaller volume of material) deforms due to overspreading (Figs 2C, 5). For hydrophobic lotus, which showed a thicker adhesive layer at the glue–lotus interface, we observed a novel behavior where tendrils or filaments of aggregate glue formed at the peaks of lotus papillae as aggregate glue pulled off from the substrate (Fig. 4B). These tendrils occurred almost exclusively on hydrophobic lotus and was mostly absent for hydrophilic lotus (observed in less than 1% of the tests) because the glue droplet has spread further and was pinned on locations of the epidermis leaf layer other than the papillae. This seems plausible due to the change in surface energy from low to high after plasma treatment where the glue tends to spread more, and less formation of a bead on a string (BOAS) morphology (e.g. Fig. 4C3) was observed.

Viscid silk may generate additional adhesive forces through physical interactions with surface lipids on the leaves, since the superhydrophobicity of lotus refers to its interactions with water. Aggregate glue is a complex cocktail of proteins, peptides, lipids and organic and inorganic compounds (Townley and Tillinghast, 2013), and future work should look at how these compounds could migrate to the surface and change the glue's wettability. However, testing this hypothesis is outside the scope of this work.

Our study found that viscid silk from spider orb webs can adhere strongly across a broad range of surface energies, important for the general diet of orb-weaving spiders like the one used in this study. Additionally, the ability of viscid silk to stick well to lotus suggests that insects have limited potential to evolve cuticular hydrophobicity as a defense against spider webs. Future investigation into the molecular basis for viscid silk's robust adhesion on both hydrophilic and superhydrophobic surfaces may help to develop tunable adhesives with multi-functional capabilities for potential applications in the biomedical, industrial, and robotic fields.

## MATERIALS AND METHODS

### Spider maintenance and silk collection

*Larinioides cornutus* (Araneae: Araneidae) orb weaving spiders were collected from two locations near Bath Township near Akron, OH, USA. Spiders were housed in cages (37 width×38 height×11.5 cm depth) and kept at The University of Akron to build webs. Spiders were fed a cricket diet after every web collection (up to three times per week), and the cages were sprayed with water every afternoon before nighttime, the time at which *L. cornutus* normally removes its web and builds a new one. *L. cornutus* viscid silk has an underlying fiber covered in evenly spaced glue droplets, 100-300 µm apart, that are around 30-40 µm in length and 20-30 µm width when measured at an intermediate humidity (55-60% RH) (Fig. 1A) (Opell and Hendricks, 2009; Opell et al., 2011). Single strands of viscid silk were collected from interior rows in the bottom side of the web, by securing strands across 12.58 mm cardboard frames using Elmer's glue (©Borden Inc.) and then cutting using a soldering iron. Samples were stored in a dark sealed box, avoiding exposure to dust and UV light, for up to 34 days until testing. Some small, non-significant, decrease in adhesion could occur on viscid silk a relatively short time (7 days) after production; however, the adhesion of long-term aged viscid silk (8-10 months) that is re-hydrated remains unchanged (Opell and Schwend, 2008). The viscid silk used in this study was rehydrated to ∼60% humidity for 5 min before and during testing and we therefore do not expect a significant effect on our results.

### Substrate preparation and surface energy testing

To test the adhesive properties of viscid silk on a superhydrophobic substrate, lotus (*Nelumbo nucifera*) leaves were used as a substrate. Lotus leaves are notable for their low surface energy and self-cleaning properties. Because of the hierarchical surface made of micro papillae and nano wax tubules, water cannot spread on the lotus leaf surface. To decouple the effect of surface chemistry from the surface topography, lotus leaves were plasma treated with oxygen for 5 min (Harrick Plasma, PDC-32G), making the leaves superhydrophilic by oxidizing the outermost surface of the leaf. Viscid silk was also tested on smooth hydrophilic clean glass as a control, as previously used in many studies (Amarpuri et al., 2015, 2017). These studies test the adhesion and spreading behavior of *L. cornutus* on smooth, hydrophilic clean glass, giving us a baseline for the silk used in the current study. Water contact angles were measured on both hydrophilic and hydrophobic lotus leaves with a Kruss Dynamic Shape Analyzer by depositing 10 μl droplets on these surfaces to provide insights into the surface energy of these substrates. Six fresh leaves from four different plants were used and three droplets for each leaf were tested for determining average water contact angles. The resulting images were analyzed using contact angle analysis plug-in with ImageJ software (Schneider et al., 2012).

### Adhesion testing

Substrates were made from the same leaves used in contact angle measurements, by adhering them to thick cardboard using double sided tape and cutting the leaves into rectangles. All substrates had the same width (5 mm) to control for the length of viscid silk coming in contact. Adhesive properties were tested using a Nano Bionix® Tensile tester (MTS Nano Instrument Innovation Center) by bringing individual threads into contact with the substrate and measuring pull-off forces. Threads were brought in contact with the substrate at a constant speed of 0.1 mm/s until a 15 nN preload, held in place for 6 s, and then pulled off until detachment using the same speed of 0.1 mm/s. Each thread was tested once on a discrete part of the substrate. All tests were done at 60±5% relative humidity, the condition at which the glue of *L. cornutus* shows maximum adhesion on glass (Amarpuri et al., 2015). Humidity was controlled inside a humidity chamber using nitrogen gas and deionized water. The work of adhesion, or the energy it takes to unstick from the substrate, was calculated as the area under the load-extension curve. Peak load during pull-off and total extension of the viscid silk were also measured. A total of 360 threads from 40 webs were tested across all three substrates (*n*=40 each for hydrophobic lotus leaves, hydrophilic lotus leaves, and glass) so that any variation among webs in stickiness was evenly distributed across treatments. Results for silk strands from the same web were averaged. Webs built by the same spider on different days were treated as independent data points. The data were checked for normality using the Shapiro–Wilk test. Because adhesion test results did not follow a normal distribution, values were log-transformed. A Wilks' lambda multivariate analysis of variance (MANOVA) was used to see if the adhesion properties differed between the treatments. Individual mixed model ANOVAs were used, with an alpha level of 0.05, to determine whether the average per web work of adhesion, peak force of adhesion and extension were different for each substrate. Each web was used as the random variable to control for inter-web differences. Tukey’s HSD was performed *post hoc* to determine pairwise differences among groups per variable. All statistical testing and graphing were performed using the JMP® Pro 17 software (©SAS Institute Inc.).

### Imaging

A scanning electron microscope [SEM; Model JEOL-7401 Japan Electron Optics Laboratory (JEOL)] was used to visualize (1) the morphology of the lotus, (2) the glue sticking behavior on the lotus leaf, and (3) the lotus substrates after adhesion tests. Samples used in (1) and (2) were observed in their native state without any sputter coating, in high vacuum operating at 1-3 kV, and were also visualized using a Keyence© VHX-7000 digital microscope (Keyence Corporation of America). Fragments of two leaves were used to observe the morphology of lotus before and after the oxygen plasma treatment. For (3), all substrates used in adhesion testing were visualized after testing to see if any glue residue was left behind. Samples were sputter coated for 45 s with gold and observed in high vacuum operating at 1-3 kV. Any aggregate glue residue left behind on the leaves was recorded. A percentage of aggregate glue residue was calculated for each lotus substrate by dividing the number of threads with aggregate glue left behind by the total number of threads tested in that substrate.

To visualize the pull-off behavior of droplets and their geometry, we looked at the viscid silk threads detaching from each of the substrates under a light microscope (Olympus BX53, EVIDENT). A 10 mm piece of viscid silk was suspended over a glass fork, and it was then brought into contact with the substrate (glass, hydrophilic lotus, or hydrophobic lotus), held in place for 6 s and then pulled away from the substrate at a constant speed and constant humidity, mimicking the same conditions as the ones used in adhesion testing (see above). Side-view photos of the pull-off were taken to see the droplet detachment using a Photron SA4 (Photron, Tokyo, Japan). The last image before full detachment was selected. Tests were repeated three times for each substrate, using viscid silk thread from three different webs. We quantified the width of all droplets in frame, at the attachment to the flagelliform axial thread, the narrowest part in the middle of the stretched droplet, and the substrate attachment, the droplet extension length, and the distance from the substrate at which the narrowest part of the glue droplet was located. We calculated asymmetry of the droplet by looking at the difference between the middle of the extended droplet (represented by zero) and the position of the narrowest part. A negative number indicates that the narrowest part is below the middle closer to the substrate. Strain was calculated as the difference between the measured droplet length extension at a given point and the original suspended droplet (pre-loaded) length divided by the original length. ImageJ was used for all of the above (Schneider et al., 2012).

## Supplementary Material

10.1242/biolopen.061802_sup1 Supplementary information
