# Supplementary material for: Viscid silk in spider orb webs adheres strongly across surfaces with different roughnesses and surface energies
Source: Biol Open. 2025 May 6;14(5):bio061802. doi: 10.1242/bio.061802 (PMC12079571; doi:10.1242/bio.061802)
Supplement: Supplementary information [file biolopen-14-061802-s1.pdf]

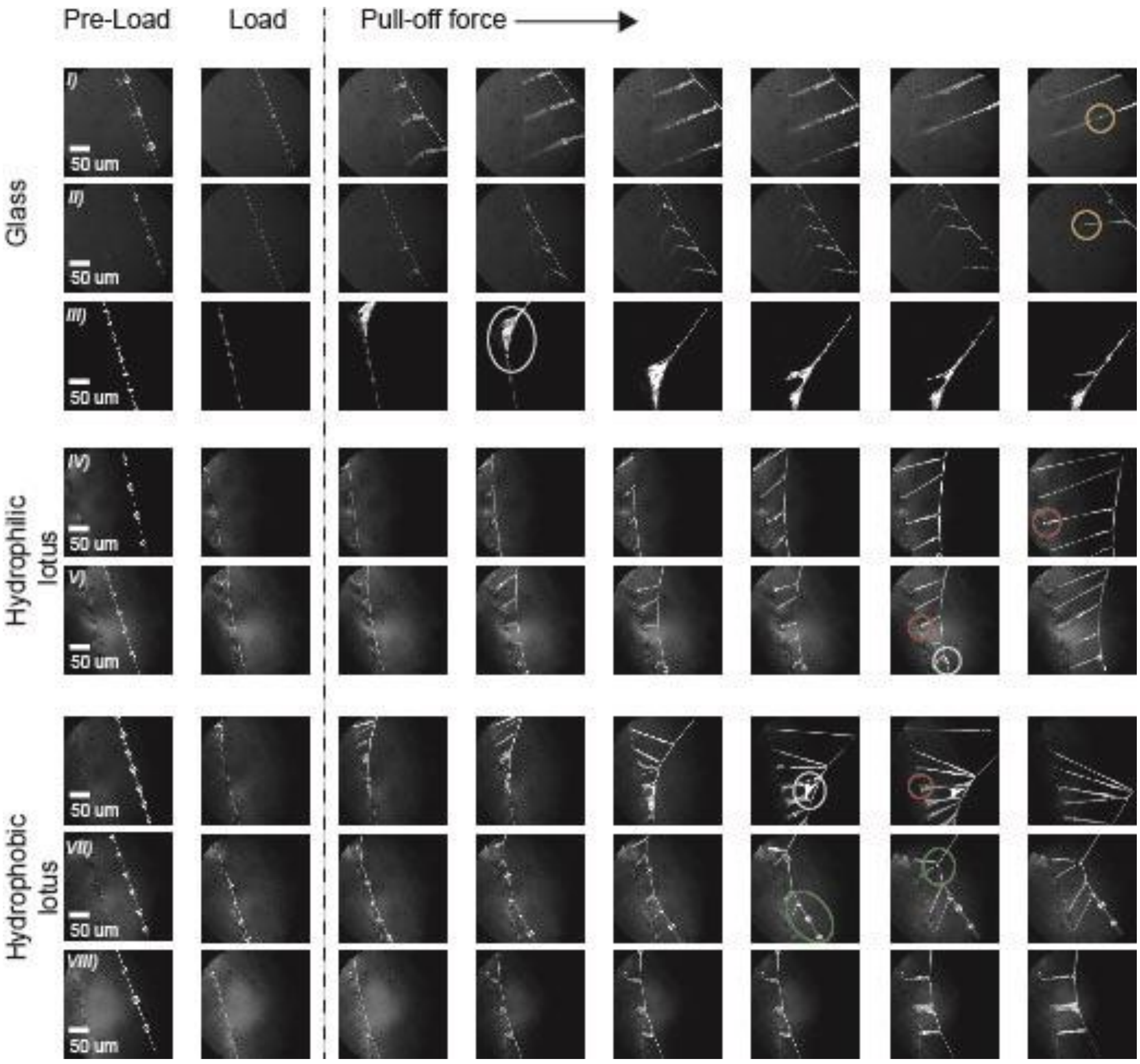


**Fig. S1.** Pre-load, load and pull off snapshots of viscid silk from glass, hydrophilic lotus and hydrophobic lotus. Each row represents still images in sequence from a single pull off event. They are arranged chronologically from left to right but are at varying time intervals to best show key events in the sequence. The first two frames show the capture thread being pushed onto the sample while the six frames to the right show the thread being pulled off the substrate, hence total adhesive force increases toward the far right. Snapshots of the last frames before total pull-off for glass (Fig. S1II),

Biology Open • Supplementary information

superhydrophobic lotus (Fig. S1VI), and hydrophilic lotus (Fig. S1IV) are also shown in Figure 5. This test was repeated three times for each substrate, using viscid silk thread from three different webs. Yellow circles suggest an interfacial failure where the glue narrowest part is located. White circles indicate instances of coalescence of droplets. Measurements at these points were not possible, and test III was excluded completely. Red circles Indicate wide attachments at the glue-substrate interface suggesting failure of glue at the bulk where the narrowest part of the extended glue was located. Green circles show droplets that did not adhere to the substrate probably because of the height/roughness variation of the lotus substrate.

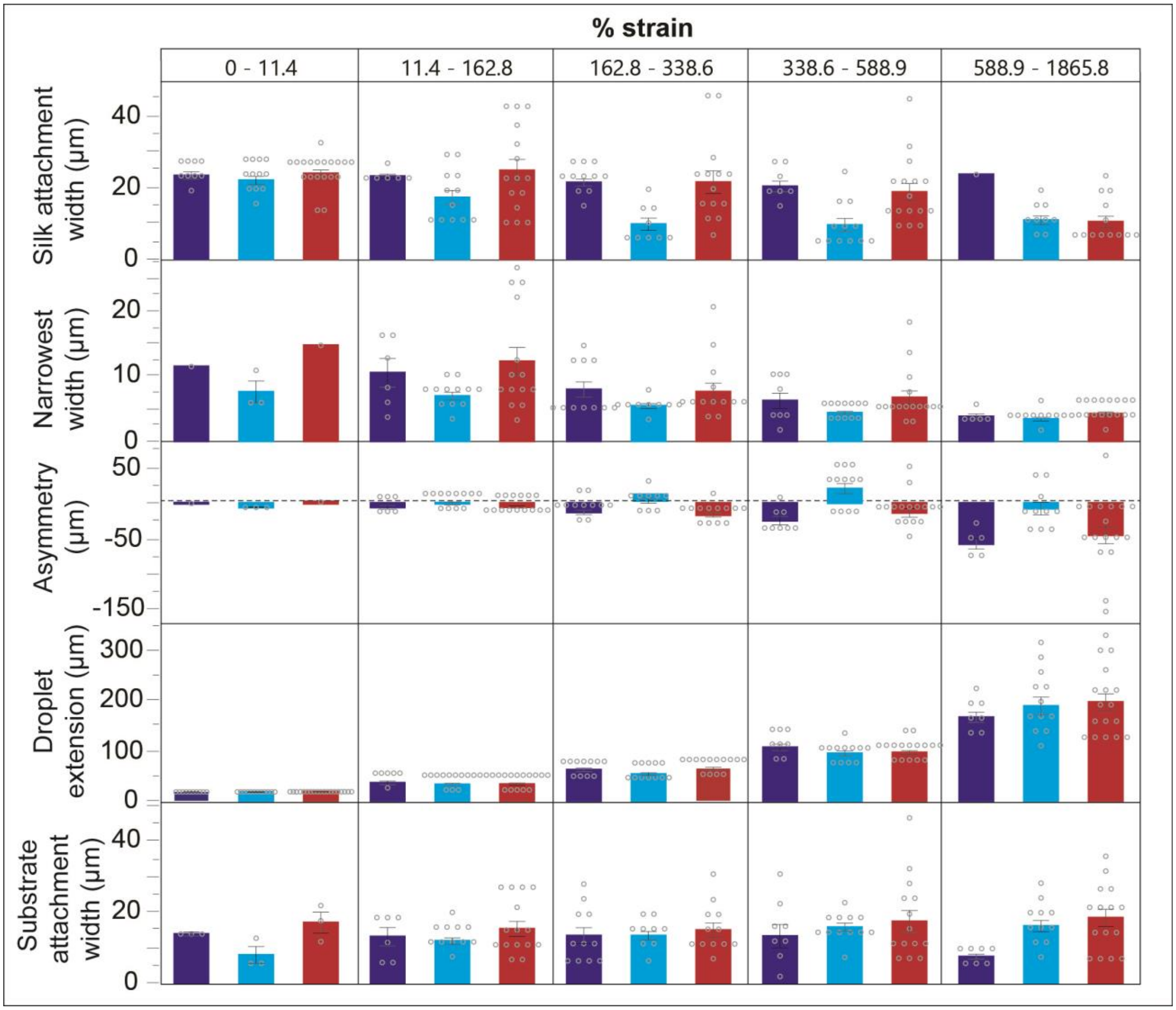


**Fig. S2.** Droplet measurements at different strains. We measured the widths at the glue-silk attachment, the glue-substrate attachment and the narrowest part of the extended droplet. We also measured extension or droplet length to calculate strains, and the asymmetry of the droplet, which refers to the position of the narrowest part of the droplet in respect to the droplet's true center. Bars represent the mean of all droplets measured in frame from 2-3 strands. Error bars represent the standard error. This graph is meant to be a visual representation of the measurements, and no statistical tests were performed because of the small sample size.

Biology Open • Supplementary information

**Table S1.** Mechanical data of viscid capture silk pull-off testing for three substrates: glass, raw lotus and hydrophilic lotus. Values represent mean of three replicates per web.

| Treatment | Spider and Web # | Peak Load (uN) | Peak Load (ln uN) | Work of Adhesion (J) | Work of Adhesion (nJ) | Work of Adhesion (ln nJ) | Extension (mm) | Extension (ln mm) |
|---|---|---|---|---|---|---|---|---|
| Philic Lotus | La.co07.01 | 970.09 | 6.88 | 3.30E-06 | 3300.00 | 8.10 | 11.73 | 2.46 |
| Philic Lotus | La.co100.01 | 1163.47 | 7.06 | 6.20E-06 | 6200.00 | 8.73 | 13.37 | 2.59 |
| Philic Lotus | La.co102.01 | 941.36 | 6.85 | 5.57E-06 | 5566.67 | 8.62 | 16.57 | 2.81 |
| Philic Lotus | La.co106.01 | 1034.29 | 6.94 | 7.24E-06 | 7242.86 | 8.89 | 18.53 | 2.92 |
| Philic Lotus | La.co111.01 | 555.44 | 6.32 | 4.50E-06 | 4500.00 | 8.41 | 19.76 | 2.98 |
| Philic Lotus | La.co112.01 | 817.62 | 6.71 | 4.60E-06 | 4600.00 | 8.43 | 12.48 | 2.52 |
| Philic Lotus | La.co112.02 | 1184.81 | 7.08 | 8.30E-06 | 8300.00 | 9.02 | 16.57 | 2.81 |
| Philic Lotus | La.co118.01 | 657.44 | 6.49 | 3.57E-06 | 3566.67 | 8.18 | 14.66 | 2.69 |
| Philic Lotus | La.co119.01 | 555.15 | 6.32 | 2.63E-06 | 2633.33 | 7.88 | 11.39 | 2.43 |
| Philic Lotus | La.co12.01 | 186.39 | 5.23 | 3.60E-07 | 360.00 | 5.89 | 7.01 | 1.95 |
| Philic Lotus | La.co127.01 | 491.21 | 6.20 | 1.90E-06 | 1895.00 | 7.55 | 9.88 | 2.29 |
| Philic Lotus | La.co128.01 | 885.47 | 6.79 | 6.17E-06 | 6166.67 | 8.73 | 18.97 | 2.94 |
| Philic Lotus | La.co129.01 | 914.12 | 6.82 | 4.77E-06 | 4766.67 | 8.47 | 12.97 | 2.56 |
| Philic Lotus | La.co134.01 | 460.31 | 6.13 | 3.77E-06 | 3766.67 | 8.23 | 21.74 | 3.08 |
| Philic Lotus | La.co137.01 | 560.17 | 6.33 | 2.63E-06 | 2633.33 | 7.88 | 12.32 | 2.51 |
| Philic Lotus | La.co20.01 | 288.74 | 5.67 | 4.57E-07 | 456.67 | 6.12 | 4.54 | 1.51 |
| Philic Lotus | La.co21.01 | 734.92 | 6.60 | 1.53E-06 | 1533.33 | 7.34 | 6.38 | 1.85 |
| Philic Lotus | La.co21a.01 | 560.46 | 6.33 | 3.23E-06 | 3233.33 | 8.08 | 16.10 | 2.78 |
| Philic Lotus | La.co21a.02 | 601.02 | 6.40 | 2.40E-06 | 2400.00 | 7.78 | 10.81 | 2.38 |
| Philic Lotus | La.co23.01 | 807.07 | 6.69 | 5.42E-06 | 5416.67 | 8.60 | 15.33 | 2.73 |
| Philic Lotus | La.co50.01 | 782.62 | 6.66 | 2.73E-06 | 2733.33 | 7.91 | 8.84 | 2.18 |
| Philic Lotus | La.co54.01 | 296.23 | 5.69 | 8.30E-07 | 829.50 | 6.72 | 7.63 | 2.03 |
| Philic Lotus | La.co55.01 | 465.33 | 6.14 | 1.93E-06 | 1933.33 | 7.57 | 9.67 | 2.27 |
| Philic Lotus | La.co55.03 | 639.19 | 6.46 | 2.13E-06 | 2133.33 | 7.67 | 9.26 | 2.23 |
| Philic Lotus | La.co55.04 | 350.40 | 5.86 | 1.43E-06 | 1433.33 | 7.27 | 11.32 | 2.43 |

| | | | | | | | | |
|---|---|---|---|---|---|---|---|---|
| Philic Lotus | La.co55.05 | 607.06 | 6.41 | 3.10E-06 | 3100.00 | 8.04 | 14.61 | 2.68 |
| Philic Lotus | La.co61.01 | 1180.71 | 7.07 | 5.83E-06 | 5833.33 | 8.67 | 13.40 | 2.60 |
| Philic Lotus | La.co61.02 | 1023.08 | 6.93 | 4.90E-06 | 4900.00 | 8.50 | 10.01 | 2.30 |
| Philic Lotus | La.co61.03 | 361.49 | 5.89 | 1.13E-06 | 1126.67 | 7.03 | 8.72 | 2.17 |
| Philic Lotus | La.co62.02 | 1118.65 | 7.02 | 6.50E-06 | 6500.00 | 8.78 | 14.30 | 2.66 |
| Philic Lotus | La.co64.01 | 1067.02 | 6.97 | 9.27E-06 | 9266.67 | 9.13 | 23.05 | 3.14 |
| Philic Lotus | La.co66.01 | 532.76 | 6.28 | 2.07E-06 | 2066.67 | 7.63 | 10.50 | 2.35 |
| Philic Lotus | La.co67.01 | 540.15 | 6.29 | 1.90E-06 | 1900.00 | 7.55 | 8.76 | 2.17 |
| Philic Lotus | La.co69.01 | 857.19 | 6.75 | 2.73E-06 | 2733.33 | 7.91 | 9.40 | 2.24 |
| Philic Lotus | La.co69.04 | 633.04 | 6.45 | 2.60E-06 | 2600.00 | 7.86 | 11.27 | 2.42 |
| Philic Lotus | La.co70.01 | 1310.03 | 7.18 | 7.20E-06 | 7200.00 | 8.88 | 15.17 | 2.72 |
| Philic Lotus | La.co70.03 | 991.60 | 6.90 | 5.40E-06 | 5400.00 | 8.59 | 16.96 | 2.83 |
| Philic Lotus | La.co71.01 | 1071.98 | 6.98 | 4.43E-06 | 4433.33 | 8.40 | 10.49 | 2.35 |
| Philic Lotus | La.co71.02 | 709.83 | 6.57 | 2.67E-06 | 2666.67 | 7.89 | 9.96 | 2.30 |
| Philic Lotus | La.coGH.03 | 882.22 | 6.78 | 5.57E-06 | 5566.67 | 8.62 | 15.77 | 2.76 |
| Raw Lotus | La.co07.01 | 1146.12 | 7.04 | 4.80E-06 | 4800.00 | 8.48 | 14.06 | 2.64 |
| Raw Lotus | La.co100.01 | 903.61 | 6.81 | 4.46E-06 | 4456.75 | 8.40 | 11.45 | 2.44 |
| Raw Lotus | La.co102.01 | 564.86 | 6.34 | 2.71E-06 | 2712.50 | 7.91 | 14.41 | 2.67 |
| Raw Lotus | La.co106.01 | 1121.35 | 7.02 | 7.43E-06 | 7425.00 | 8.91 | 19.23 | 2.96 |
| Raw Lotus | La.co111.01 | 573.51 | 6.35 | 3.73E-06 | 3733.33 | 8.23 | 19.32 | 2.96 |
| Raw Lotus | La.co112.01 | 1239.09 | 7.12 | 5.83E-06 | 5833.33 | 8.67 | 13.85 | 2.63 |
| Raw Lotus | La.co112.02 | 1670.27 | 7.42 | 1.32E-05 | 13200.00 | 9.49 | 20.69 | 3.03 |
| Raw Lotus | La.co118.01 | 1094.85 | 7.00 | 6.77E-06 | 6766.67 | 8.82 | 20.34 | 3.01 |
| Raw Lotus | La.co119.01 | 594.33 | 6.39 | 2.73E-06 | 2733.33 | 7.91 | 12.05 | 2.49 |
| Raw Lotus | La.co12.01 | 210.77 | 5.35 | 5.10E-07 | 510.00 | 6.23 | 7.74 | 2.05 |
| Raw Lotus | La.co127.01 | 1136.69 | 7.04 | 4.50E-06 | 4500.00 | 8.41 | 13.09 | 2.57 |
| Raw Lotus | La.co128.01 | 864.84 | 6.76 | 5.37E-06 | 5366.67 | 8.59 | 17.10 | 2.84 |
| Raw Lotus | La.co129.01 | 846.99 | 6.74 | 6.70E-06 | 6700.00 | 8.81 | 18.84 | 2.94 |
| Raw Lotus | La.co134.01 | 366.51 | 5.90 | 1.73E-06 | 1733.33 | 7.46 | 15.03 | 2.71 |
| Raw Lotus | La.co137.01 | 645.60 | 6.47 | 2.87E-06 | 2866.67 | 7.96 | 13.20 | 2.58 |
| Raw Lotus | La.co20.01 | 692.95 | 6.54 | 1.46E-06 | 1460.00 | 7.29 | 7.62 | 2.03 |

Biology Open • Supplementary information

| | | | | | | | | |
|---|---|---|---|---|---|---|---|---|
| Raw Lotus | La.co21.01 | 1046.27 | 6.95 | 2.60E-06 | 2600.00 | 7.86 | 7.81 | 2.05 |
| Raw Lotus | La.co21a.01 | 540.04 | 6.29 | 5.58E-06 | 5577.50 | 8.63 | 22.62 | 3.12 |
| Raw Lotus | La.co21a.02 | 685.37 | 6.53 | 2.53E-06 | 2533.33 | 7.84 | 10.22 | 2.32 |
| Raw Lotus | La.co23.01 | 906.87 | 6.81 | 4.36E-06 | 4361.25 | 8.38 | 14.29 | 2.66 |
| Raw Lotus | La.co50.01 | 1551.30 | 7.35 | 7.23E-06 | 7233.33 | 8.89 | 11.96 | 2.48 |
| Raw Lotus | La.co54.01 | 560.82 | 6.33 | 2.23E-06 | 2233.33 | 7.71 | 13.73 | 2.62 |
| Raw Lotus | La.co55.01 | 692.73 | 6.54 | 2.13E-06 | 2133.33 | 7.67 | 13.22 | 2.58 |
| Raw Lotus | La.co55.03 | 1037.17 | 6.94 | 5.20E-06 | 5200.00 | 8.56 | 12.90 | 2.56 |
| Raw Lotus | La.co55.04 | 845.15 | 6.74 | 3.90E-06 | 3900.00 | 8.27 | 13.88 | 2.63 |
| Raw Lotus | La.co55.05 | 534.46 | 6.28 | 2.40E-06 | 2400.00 | 7.78 | 15.18 | 2.72 |
| Raw Lotus | La.co61.01 | 1569.73 | 7.36 | 1.28E-05 | 12833.33 | 9.46 | 21.95 | 3.09 |
| Raw Lotus | La.co61.02 | 1141.34 | 7.04 | 5.17E-06 | 5166.67 | 8.55 | 13.96 | 2.64 |
| Raw Lotus | La.co61.03 | 932.43 | 6.84 | 1.07E-05 | 10733.33 | 9.28 | 27.24 | 3.30 |
| Raw Lotus | La.co62.02 | 1714.66 | 7.45 | 9.10E-06 | 9100.00 | 9.12 | 15.76 | 2.76 |
| Raw Lotus | La.co64.01 | 897.67 | 6.80 | 7.05E-06 | 7050.00 | 8.86 | 22.45 | 3.11 |
| Raw Lotus | La.co66.01 | 689.63 | 6.54 | 5.45E-06 | 5450.00 | 8.60 | 21.04 | 3.05 |
| Raw Lotus | La.co67.01 | 850.92 | 6.75 | 7.25E-06 | 7250.00 | 8.89 | 21.04 | 3.05 |
| Raw Lotus | La.co69.01 | 773.47 | 6.65 | 2.07E-06 | 2066.67 | 7.63 | 8.81 | 2.18 |
| Raw Lotus | La.co69.04 | 634.72 | 6.45 | 2.30E-06 | 2300.00 | 7.74 | 10.52 | 2.35 |
| Raw Lotus | La.co70.01 | 1516.15 | 7.32 | 1.49E-05 | 14850.00 | 9.61 | 27.30 | 3.31 |
| Raw Lotus | La.co70.03 | 1359.28 | 7.21 | 8.07E-06 | 8066.67 | 9.00 | 19.21 | 2.96 |
| Raw Lotus | La.co71.01 | 794.80 | 6.68 | 2.14E-06 | 2142.50 | 7.67 | 8.74 | 2.17 |
| Raw Lotus | La.co71.02 | 904.43 | 6.81 | 2.93E-06 | 2933.33 | 7.98 | 10.10 | 2.31 |
| Raw Lotus | La.coGH.03 | 852.08 | 6.75 | 7.08E-06 | 7075.00 | 8.86 | 19.24 | 2.96 |
| Glass | La.co07.01 | 644.47 | 6.47 | 2.53E-06 | 2533.33 | 7.84 | 11.22 | 2.42 |
| Glass | La.co100.01 | 336.44 | 5.82 | 9.50E-07 | 950.00 | 6.86 | 8.34 | 2.12 |
| Glass | La.co102.01 | 495.71 | 6.21 | 2.60E-06 | 2600.00 | 7.86 | 15.42 | 2.74 |
| Glass | La.co106.01 | 407.81 | 6.01 | 1.85E-06 | 1846.67 | 7.52 | 13.95 | 2.64 |
| Glass | La.co111.01 | 541.27 | 6.29 | 2.33E-06 | 2333.33 | 7.76 | 15.53 | 2.74 |
| Glass | La.co112.01 | 327.89 | 5.79 | 1.33E-06 | 1330.00 | 7.19 | 10.24 | 2.33 |
| Glass | La.co112.02 | 202.95 | 5.31 | 7.37E-07 | 736.67 | 6.60 | 8.84 | 2.18 |

| | | | | | | | | |
|---|---|---|---|---|---|---|---|---|
| Glass | La.co118.01 | 211.05 | 5.35 | 8.30E-07 | 830.00 | 6.72 | 10.98 | 2.40 |
| Glass | La.co119.01 | 540.60 | 6.29 | 1.83E-06 | 1833.33 | 7.51 | 9.78 | 2.28 |
| Glass | La.co12.01 | 306.10 | 5.72 | 1.02E-06 | 1020.00 | 6.93 | 9.45 | 2.25 |
| Glass | La.co127.01 | 144.13 | 4.97 | 3.77E-07 | 376.67 | 5.93 | 5.81 | 1.76 |
| Glass | La.co128.01 | 264.83 | 5.58 | 1.10E-06 | 1100.00 | 7.00 | 12.82 | 2.55 |
| Glass | La.co129.01 | 228.92 | 5.43 | 7.47E-07 | 746.67 | 6.62 | 8.13 | 2.10 |
| Glass | La.co134.01 | 202.25 | 5.31 | 9.77E-07 | 976.67 | 6.88 | 14.44 | 2.67 |
| Glass | La.co137.01 | 471.87 | 6.16 | 1.97E-06 | 1966.67 | 7.58 | 12.37 | 2.51 |
| Glass | La.co20.01 | 631.51 | 6.45 | 1.57E-06 | 1566.67 | 7.36 | 7.23 | 1.98 |
| Glass | La.co21.01 | 965.57 | 6.87 | 2.90E-06 | 2900.00 | 7.97 | 10.42 | 2.34 |
| Glass | La.co21a.01 | 272.56 | 5.61 | 1.00E-06 | 1003.33 | 6.91 | 9.34 | 2.23 |
| Glass | La.co21a.02 | 318.74 | 5.76 | 1.39E-06 | 1390.00 | 7.24 | 11.81 | 2.47 |
| Glass | La.co23.01 | 388.92 | 5.96 | 1.44E-06 | 1436.67 | 7.27 | 10.49 | 2.35 |
| Glass | La.co50.01 | 249.94 | 5.52 | 6.93E-07 | 693.33 | 6.54 | 7.02 | 1.95 |
| Glass | La.co54.01 | 190.95 | 5.25 | 6.20E-07 | 620.00 | 6.43 | 9.23 | 2.22 |
| Glass | La.co55.01 | 516.86 | 6.25 | 1.65E-06 | 1645.00 | 7.41 | 8.33 | 2.12 |
| Glass | La.co55.03 | 187.46 | 5.23 | 5.30E-07 | 530.00 | 6.27 | 7.23 | 1.98 |
| Glass | La.co55.04 | 447.77 | 6.10 | 1.63E-06 | 1633.33 | 7.40 | 12.40 | 2.52 |
| Glass | La.co55.05 | 316.27 | 5.76 | 1.60E-06 | 1600.00 | 7.38 | 12.97 | 2.56 |
| Glass | La.co61.01 | 317.08 | 5.76 | 1.33E-06 | 1333.33 | 7.20 | 11.47 | 2.44 |
| Glass | La.co61.02 | 395.18 | 5.98 | 1.10E-06 | 1096.67 | 7.00 | 7.98 | 2.08 |
| Glass | La.co61.03 | 336.16 | 5.82 | 9.23E-07 | 923.33 | 6.83 | 8.20 | 2.10 |
| Glass | La.co62.02 | 341.31 | 5.83 | 1.36E-06 | 1355.00 | 7.21 | 10.57 | 2.36 |
| Glass | La.co66.01 | 199.56 | 5.30 | 5.63E-07 | 563.33 | 6.33 | 7.07 | 1.96 |
| Glass | La.co67.01 | 355.50 | 5.87 | 9.85E-07 | 985.00 | 6.89 | 8.69 | 2.16 |
| Glass | La.co69.01 | 166.64 | 5.12 | 3.63E-07 | 363.33 | 5.90 | 5.58 | 1.72 |
| Glass | La.co69.04 | 296.69 | 5.69 | 8.33E-07 | 833.33 | 6.73 | 7.77 | 2.05 |
| Glass | La.co70.01 | 838.01 | 6.73 | 3.30E-06 | 3300.00 | 8.10 | 15.19 | 2.72 |
| Glass | La.co70.03 | 464.88 | 6.14 | 2.40E-06 | 2400.00 | 7.78 | 14.62 | 2.68 |
| Glass | La.co71.01 | 369.35 | 5.91 | 1.30E-06 | 1300.00 | 7.17 | 10.40 | 2.34 |
| Glass | La.co71.02 | 162.85 | 5.09 | 6.43E-07 | 643.33 | 6.47 | 6.91 | 1.93 |
| Glass | La.coGH.03 | 316.27 | 5.76 | 1.60E-06 | 1600.00 | 7.38 | 11.93 | 2.48 |
| Glass | La.co64 | 610.07 | 6.41 | 2.47E-06 | 2466.67 | 7.81 | 13.25 | 2.58 |

**Table S2.** Contact angle measurements of water on the testing surfaces.

| Treatment | Substrate ID | Leaf # | Contact Angle |
|---|---|---|---|
| Raw Lotus | R3A | 3 | 144.1 |
| Raw Lotus | R3A | 3 | 131.2 |
| Raw Lotus | R3A | 3 | 146.1 |
| Raw Lotus | R4A | 4 | 145.2 |
| Raw Lotus | R4A | 4 | 140.9 |
| Raw Lotus | R4A | 4 | 145.4 |
| Raw Lotus | R4B | 4 | 145.5 |
| Raw Lotus | R4B | 4 | 144.9 |
| Raw Lotus | R4B | 4 | 141.5 |
| Raw Lotus | R6A | 6 | 138.7 |
| Raw Lotus | R6A | 6 | 147.1 |
| Raw Lotus | R6A | 6 | 152.1 |
| Raw Lotus | R7A | 7 | 147.1 |
| Raw Lotus | R7A | 7 | 143.4 |
| Raw Lotus | R7A | 7 | 140.4 |

*Philic and glass substrates had a contact angle of zero

**Table S3.** Cohesive failure and behavior of glue on tested lotus substrates: hydrophobic (raw) and hydrophilic (philic).

| Treatment | Substrate ID | Leaf # | Total samples tested | Cohesive failure | Cohesive failure (%) | Tendrils | Comments |
|---|---|---|---|---|---|---|---|
| Philic Lotus | PT3A | 3 | 9 | 9 | 100 | Yes (1) | |
| Philic Lotus | PT3B | 3 | 9 | 9 | 100 | Yes (3) | Thread left behind |
| Philic Lotus | PT4A | 4 | 12 | 1 | 8 | No | Thread left behind |
| Philic Lotus | PT4B | 4 | 9 | 2 | 22 | No | |
| Philic Lotus | PT7A | 7 | 9 | 0 | 0 | No | |
| Philic Lotus | PT7B | 7 | 9 | No SEM | | NA | |
| Philic Lotus | PT7C | 7 | 11 | 4 | 36 | No | |
| Philic Lotus | PT7D | 7 | 13 | No SEM | | NA | |
| Philic Lotus | PT7E | 7 | 9 | 5 | 56 | No | |
| Philic Lotus | PT8A | 8 | 9 | 0 | 0 | 0 | |
| Philic Lotus | PT8B | 8 | 9 | 6 | 67 | 0 | |
| Philic Lotus | PT8C | 8 | 9 | No SEM | | NA | |
| Raw Lotus | R3A | 3 | 10 | 10 | 100 | Yes | |
| Raw Lotus | R3B | 3 | 11 | 8 | 73 | Yes | |
| Raw Lotus | R4A | 4 | 10 | 2 | 20 | Yes | |
| Raw Lotus | R4B | 4 | 10 | 3 | 30 | Yes | |
| Raw Lotus | R7A | 7 | 10 | 0 | 0 | Yes | |
| Raw Lotus | R7B | 7 | 10 | 5 | 50 | Yes | |
| Raw Lotus | R7D | 7 | 9 | 2 | 22 | Yes | |
| Raw Lotus | R7E | 7 | 11 | 1 | 9 | Yes | |
| Raw Lotus | R7F | 7 | 14 | No SEM | | NA | |
| Raw Lotus | R8A | 8 | 10 | 4 | 40 | Yes | |
| Raw Lotus | R8B | 8 | 10 | 0 | 0 | Yes | |
| Raw Lotus | R8C | 8 | 10 | 10 | 100 | Yes | Thread left behind |

**Table S4.** Pull off measurement data of viscid capture silk for three substrates: glass, hydrophobic raw lotus (phobic) and hydrophilic lotus. Two to three capture silk strands were tested per substrate. All droplets in-frame were measured. Picture order 0 represents the pre-load suspended samples, 1 represents the loaded spread sample, and 7 represents the last picture right before detachment. Some measurements were difficult to obtain and therefore we kept the cells empty.

| Treatment | Sample | Name on figure S1 | Picture order | Droplet # | Width silk-glue (um) | Width middle (um) | Width thinnest part (um) | % change of thinnest part from suspended droplet | Width substrate-glue (um) | Height of thinnest part (um) | Droplet pulled length (um) | % strain |
|---|---|---|---|---|---|---|---|---|---|---|---|---|
| Glass | La.co01.02 | I | 0 | 1 | 25.885 | | | | | | 20.292 | 0 |
| Glass | La.co01.02 | I | 1 | 1 | | | | | | | | |
| Glass | La.co01.02 | I | 2 | 1 | 14.836 | 14.539 | 12.868 | 50.287811 | 25.75 | 43.83 | 68.449 | 237.32013 |
| Glass | La.co01.02 | I | 3 | 1 | 14.794 | 16.128 | 7.99 | 69.132702 | 30.661 | 69.342 | 132.643 | 553.6714 |
| Glass | La.co01.02 | I | 4 | 1 | | 9.051 | | | 9.306 | | 168.067 | 728.24266 |
| Glass | La.co01.02 | I | 5 | 1 | | | | | 5.964 | | 178.887 | 781.56416 |
| Glass | La.co01.02 | I | 6 | 1 | | | | | | | | |
| Glass | La.co01.02 | I | 7 | 1 | | | | | | | | |
| Glass | La.co01.02 | I | 0 | 2 | 27.048 | | | | | | 21.789 | 0 |
| Glass | La.co01.02 | I | 1 | 2 | | | | | | | | |
| Glass | La.co01.02 | I | 2 | 2 | 22.248 | 16.745 | 16.317 | 39.673913 | 19.364 | 19.225 | 40.909 | 87.7507 |
| Glass | La.co01.02 | I | 3 | 2 | 18.669 | 12.915 | 12.159 | 55.046584 | 23.658 | 40.036 | 79.728 | 265.9094 |
| Glass | La.co01.02 | I | 4 | 2 | 18.331 | 10.53 | 10.216 | 62.230109 | 13.807 | 35.795 | 109.171 | 401.03722 |
| Glass | La.co01.02 | I | 5 | 2 | 17.096 | 10.196 | 9.367 | 65.368974 | 12.905 | 26.182 | 117.166 | 437.73005 |
| Glass | La.co01.02 | I | 6 | 2 | | 10.112 | 5.22 | 80.700976 | 9.334 | 22.361 | 176.683 | 710.88164 |
| Glass | La.co01.02 | I | 7 | 2 | | 6.806 | 3.311 | 87.758799 | 7.898 | 23.504 | 209.769 | 862.7289 |
| Glass | La.co01.02 | I | 0 | 3 | 25.799 | | | | | | 20.931 | 0 |
| Glass | La.co01.02 | I | 1 | 3 | | | | | | | | |

Biology Open • Supplementary information

| | | | | | | | | | | | | |
|---|---|---|---|---|---|---|---|---|---|---|---|---|
| Glass | La.co01.02 | I | 2 | 3 | 20.998 | 17.555 | 16.127 | 37.489825 | 18.32 | 10.762 | 25.531 | 21.976972 |
| Glass | La.co01.02 | I | 3 | 3 | 23.254 | 12.924 | 12.73 | 50.657002 | 17.401 | 27.531 | 50.307 | 140.34685 |
| Glass | La.co01.02 | I | 4 | 3 | 21.916 | 13.281 | 13.8 | | 19.293 | 14.115 | 69.104 | 230.15145 |
| Glass | La.co01.02 | I | 5 | 3 | 21.722 | 12.389 | 12.126 | 52.998178 | 19.343 | 32.336 | 76.214 | 264.1202 |
| Glass | La.co01.02 | I | 6 | 3 | | 11.51 | 9.023 | 65.025776 | 22.275 | 55.362 | 123.923 | 492.05485 |
| Glass | La.co01.02 | I | 7 | 3 | | | 3.995 | 84.514904 | 8.474 | 26.622 | 158.209 | 655.85973 |
| Phobic | La.co02.01 | VII | 0 | 1 | 24.314 | | | | | | 18.979 | 0 |
| Phobic | La.co02.01 | VII | 1 | 1 | | | | | | | | |
| Phobic | La.co02.01 | VII | 2 | 1 | | | | | | | | |
| Phobic | La.co02.01 | VII | 3 | 1 | | | | | | | | |
| Phobic | La.co02.01 | VII | 4 | 1 | | | | | | | | |
| Phobic | La.co02.01 | VII | 5 | 1 | | | | | | | | |
| Phobic | La.co02.01 | VII | 6 | 1 | | | | | | | | |
| Phobic | La.co02.01 | VII | 7 | 1 | | | | | | | | |
| Phobic | La.co02.01 | VII | 0 | 2 | 22.305 | | | | | | 16.967 | 0 |
| Phobic | La.co02.01 | VII | 1 | 2 | | | | | | | | |
| Phobic | La.co02.01 | VII | 2 | 2 | 20.706 | 17.907 | | | 8.63 | | 19.528 | 15.094006 |
| Phobic | La.co02.01 | VII | 3 | 2 | 17.627 | 10.535 | 3.244 | 85.456176 | 5.749 | 7.101 | 31.434 | 85.265515 |
| Phobic | La.co02.01 | VII | 4 | 2 | 16.608 | 4.048 | 3.476 | 84.41605 | 10.351 | 20.296 | 49.082 | 189.27919 |
| Phobic | La.co02.01 | VII | 5 | 2 | 14.859 | 3.675 | 2.105 | | 5.907 | 6.796 | 84.072 | 395.50304 |
| Phobic | La.co02.01 | VII | 6 | 2 | 18.133 | 3.059 | 2.847 | 87.236046 | | 2.097 | 120.168 | 608.24542 |
| Phobic | La.co02.01 | VII | 7 | 2 | 16.382 | 3.711 | 1.773 | 92.05111 | | 2.054 | 141.783 | 735.63977 |
| Phobic | La.co02.01 | VII | 0 | 3 | 23.121 | | | | | | 17.588 | 0 |
| Phobic | La.co02.01 | VII | 1 | 3 | | | | | | | | |
| Phobic | La.co02.01 | VII | 2 | 3 | | | | | | | | |
| Phobic | La.co02.01 | VII | 3 | 3 | 14.963 | 15.294 | 14.667 | 36.564162 | 21.749 | 10.225 | 17.626 | 0.2160564 |
| Phobic | La.co02.01 | VII | 4 | 3 | 11.435 | 13.259 | 10.146 | 56.117815 | 12.744 | 7.84 | 34.537 | 96.366841 |
| Phobic | La.co02.01 | VII | 5 | 3 | 11.696 | 7.558 | 5.059 | 78.119459 | 18.806 | 10.336 | 66.495 | 278.07028 |
| Phobic | La.co02.01 | VII | 6 | 3 | 13.379 | 5.525 | 4.795 | 79.261278 | 12.583 | 8.732 | 97.912 | 456.69775 |
| Phobic | La.co02.01 | VII | 7 | 3 | 13.116 | 4.727 | 3.964 | 82.855413 | 11.685 | 41.055 | 109.183 | 520.78121 |
| Phobic | La.co02.01 | VII | 0 | 4 | 23.956 | | | | | | 18.563 | 0 |

| | | | | | | | | | | | | |
|---|---|---|---|---|---|---|---|---|---|---|---|---|
| Phobic | La.co02.01 | VII | 1 | 4 | | | | | | | | |
| Phobic | La.co02.01 | VII | 2 | 4 | | | | | | | | |
| Phobic | La.co02.01 | VII | 3 | 4 | | | | | | | | |
| Phobic | La.co02.01 | VII | 4 | 4 | | | | | | | | |
| Phobic | La.co02.01 | VII | 5 | 4 | | | | | | | | |
| Phobic | La.co02.01 | VII | 6 | 4 | | | | | | | | |
| Phobic | La.co02.01 | VII | 7 | 4 | | | | | | | | |
| Phobic | La.co02.01 | VII | 0 | 5 | 22.477 | | | | | | 17.138 | 0 |
| Phobic | La.co02.01 | VII | 1 | 5 | | | | | | | | |
| Phobic | La.co02.01 | VII | 2 | 5 | | | | | | | | |
| Phobic | La.co02.01 | VII | 3 | 5 | 10.413 | 10.169 | 9.369 | 58.317391 | 9.097 | 14.361 | 31.368 | 83.031859 |
| Phobic | La.co02.01 | VII | 4 | 5 | 10.951 | 8.349 | 6.119 | 72.776616 | 8.313 | 9.789 | 53.044 | 209.51103 |
| Phobic | La.co02.01 | VII | 5 | 5 | 23.405 | 6.736 | 6.052 | 73.074699 | | 15.314 | 64.659 | 277.2844 |
| Phobic | La.co02.01 | VII | 6 | 5 | 21.796 | 6.485 | 4.9 | 78.199938 | 7.645 | 21.121 | 79.668 | 364.86171 |
| Phobic | La.co02.01 | VII | 7 | 5 | 20.736 | 4.795 | 4.663 | 79.254349 | | 27.317 | 95.356 | 456.40098 |
| Phobic | La.co02.01 | VII | 0 | 6 | 22.201 | | | | | | 16.576 | 0 |
| Phobic | La.co02.01 | VII | 1 | 6 | | | | | | | | |
| Phobic | La.co02.01 | VII | 2 | 6 | 12.254 | 11.269 | | | 11.568 | | 18.462 | 11.377896 |
| Phobic | La.co02.01 | VII | 3 | 6 | 8.702 | 7.934 | 7.379 | 66.762758 | 9.647 | 27.945 | 43.037 | 159.63441 |
| Phobic | La.co02.01 | VII | 4 | 6 | 6.691 | 8.306 | 4.079 | | 10.865 | 9.745 | 62.935 | 279.67543 |
| Phobic | La.co02.01 | VII | 5 | 6 | 23.406 | 7.449 | | | 11.376 | | 70.443 | 324.96984 |
| Phobic | La.co02.01 | VII | 6 | 6 | 21.796 | 7.456 | 6.202 | 72.064321 | 9.586 | 33.137 | 85.651 | 416.71694 |
| Phobic | La.co02.01 | VII | 7 | 6 | 20.736 | 6.389 | 5.506 | 75.199315 | 8.251 | 44.17 | 102.618 | 519.07577 |
| Glass | La.co02.02 | II | 0 | 1 | 20.293 | | | | | | 16.273 | 0 |
| Glass | La.co02.02 | II | 1 | 1 | | | | | | | | |
| Glass | La.co02.02 | II | 2 | 1 | 23.711 | 10.757 | 8.08 | 60.183314 | 11.344 | 6.116 | 28.64 | 75.99705 |
| Glass | La.co02.02 | II | 3 | 1 | 21.878 | 8.179 | 4.875 | 75.976938 | 5.546 | 3.744 | 54.951 | 237.68205 |
| Glass | La.co02.02 | II | 4 | 1 | 25.009 | 6.388 | 4.034 | 80.121224 | 7.83 | 13.29 | 92.142 | 466.22626 |
| Glass | La.co02.02 | II | 5 | 1 | 18.957 | 6.281 | 3.754 | 81.50101 | 7.565 | 14.155 | 105.707 | 549.5852 |
| Glass | La.co02.02 | II | 6 | 1 | | 5.549 | 2.997 | 85.231361 | 5.464 | 22.409 | 129.44 | 695.42801 |
| Glass | La.co02.02 | II | 7 | 1 | 23.537 | 4.487 | 3.3033 | | 4.892 | 25.324 | 141.091 | 767.02513 |

| | | | | | | | | | | | | |
|---|---|---|---|---|---|---|---|---|---|---|---|---|
| Glass | La.co02.02 | II | 0 | 2 | 20.114 | | | | | | 16.827 | 0 |
| Glass | La.co02.02 | II | 1 | 2 | | | | | | | | |
| Glass | La.co02.02 | II | 2 | 2 | 21.672 | 14.594 | | | 13.691 | | 18.496 | 9.9185832 |
| Glass | La.co02.02 | II | 3 | 2 | 24.806 | 9.633 | 4.698 | 76.643134 | 5.87 | 3.449 | 46.901 | 178.72467 |
| Glass | La.co02.02 | II | 4 | 2 | 21.631 | 6.692 | 4.271 | 78.766034 | 5.482 | 9.386 | 65.958 | 291.97718 |
| Glass | La.co02.02 | II | 5 | 2 | 24.306 | 5.435 | 1.74 | 91.349309 | 1.74 | 0 | 83.454 | 395.95293 |
| Glass | La.co02.02 | II | 6 | 2 | | | | | | | | |
| Glass | La.co02.02 | II | 7 | 2 | | | | | | | | |
| Glass | La.co02.02 | II | 0 | 3 | 19.042 | | | | | | 15.982 | 0 |
| Glass | La.co02.02 | II | 1 | 3 | | | | | | | | |
| Glass | La.co02.02 | II | 2 | 3 | 22.679 | 10.854 | | | 12.827 | | 12.594 | -21.19885 |
| Glass | La.co02.02 | II | 3 | 3 | 25.368 | 9.035 | 3.69 | | 4.932 | 4.423 | 32.719 | 104.72406 |
| Glass | La.co02.02 | II | 4 | 3 | 18.073 | 7.588 | 5.767 | 69.714316 | 10.569 | 11.774 | 48.842 | 205.60631 |
| Glass | La.co02.02 | II | 5 | 3 | 25.77 | 6.562 | 5.374 | 71.778175 | 11.167 | 15.287 | 60.51 | 278.61344 |
| Glass | La.co02.02 | II | 6 | 3 | 22.931 | 5.853 | 3.4 | 82.144733 | 8.2 | 11.545 | 83.17 | 420.39795 |
| Glass | La.co02.02 | II | 7 | 3 | | | | | | | | |
| Glass | La.co02.02 | II | 0 | 4 | | | | | | | 17.027 | 0 |
| Glass | La.co02.02 | II | 1 | 4 | | | | | | | | |
| Glass | La.co02.02 | II | 2 | 4 | | | | | | | | |
| Glass | La.co02.02 | II | 3 | 4 | 26.622 | 13.794 | 11.43 | 57.065585 | 14.677 | 6.869 | 14.709 | -13.61367 |
| Glass | La.co02.02 | II | 4 | 4 | 22.594 | 10.028 | 5.887 | 77.88671 | 6.443 | 2.383 | 35.05 | 105.84953 |
| Glass | La.co02.02 | II | 5 | 4 | 24.514 | 8.747 | 5.67 | 78.701826 | 11.332 | 7.584 | 44.747 | 162.80026 |
| Glass | La.co02.02 | II | 6 | 4 | 20.266 | 7.725 | 5.265 | 80.223124 | 7.667 | 13.208 | 59.922 | 251.92342 |
| Glass | La.co02.02 | II | 7 | 4 | | | | | | | | |
| Phobic | La.co02.04 | VII | 0 | 1 | 27.218 | | | | | | 18.453 | 0 |
| Phobic | La.co02.04 | VII | 1 | 1 | | | | | | | | |
| Phobic | La.co02.04 | VII | 2 | 1 | | | | | | | 21.003 | 13.818891 |
| Phobic | La.co02.04 | VII | 3 | 1 | 19.045 | 14.689 | 8.189 | 69.913293 | 13.782 | 3.978 | 32.381 | 75.478242 |
| Phobic | La.co02.04 | VII | 4 | 1 | 23.224 | 10.796 | 7.647 | 71.904622 | 12.017 | 3.995 | 43.74 | 137.03463 |
| Phobic | La.co02.04 | VII | 5 | 1 | 13.907 | 9.217 | 5.07 | 81.372621 | 6.754 | 2.773 | 54.732 | 196.60218 |
| Phobic | La.co02.04 | VII | 6 | 1 | 20.743 | 7.831 | 4.76 | 82.511573 | | 2.359 | 72.057 | 290.48935 |

| | | | | | | | | | | | | |
|---|---|---|---|---|---|---|---|---|---|---|---|---|
| Phobic | La.co02.04 | VII | 7 | 1 | 13.213 | 7.608 | 4.406 | 83.812183 | | 1.796 | 97.359 | 427.60527 |
| Phobic | La.co02.04 | VII | 0 | 2 | 26.872 | | | | | | 18.643 | 0 |
| Phobic | La.co02.04 | VII | 1 | 2 | | | | | | | | |
| Phobic | La.co02.04 | VII | 2 | 2 | | | | | | | | |
| Phobic | La.co02.04 | VII | 3 | 2 | 37.275 | 27.082 | 25.397 | 5.4889848 | 26.416 | 5.106 | 21.025 | 12.776914 |
| Phobic | La.co02.04 | VII | 4 | 2 | 41.48 | 24.37 | 23.025 | 14.316017 | 27.067 | 14.376 | 33.324 | 78.748056 |
| Phobic | La.co02.04 | VII | 5 | 2 | 43.884 | 23.537 | 22.045 | 17.962935 | 25.972 | 22.158 | 47.503 | 154.80341 |
| Phobic | La.co02.04 | VII | 6 | 2 | 46.17 | 20.614 | 20.575 | 23.433313 | 30.633 | 25.226 | 63.953 | 243.04028 |
| Phobic | La.co02.04 | VII | 7 | 2 | 44.605 | 17.953 | 18.239 | 32.126377 | 30.266 | 29.679 | 87.607 | 369.919 |
| Phobic | La.co02.04 | VII | 0 | 3 | 24.344 | | | | | | 18.235 | 0 |
| Phobic | La.co02.04 | VII | 1 | 3 | | | | | | | | |
| Phobic | La.co02.04 | VII | 2 | 3 | 32.364 | 16.696 | | | 17.463 | | 17.646 | -3.230052 |
| Phobic | La.co02.04 | VII | 3 | 3 | 29.882 | 12.785 | 4.875 | 79.974532 | 8.546 | 7.116 | 30.899 | 69.448862 |
| Phobic | La.co02.04 | VII | 4 | 3 | 21.682 | 10.507 | 4.755 | 80.467466 | 11.192 | 11.304 | 46.539 | 155.21799 |
| Phobic | La.co02.04 | VII | 5 | 3 | 23.916 | 9.749 | 5.883 | | 15.023 | 18.545 | 61.186 | 235.54154 |
| Phobic | La.co02.04 | VII | 6 | 3 | 28.248 | 8.253 | 6.355 | | 8.496 | 14.307 | 79.969 | 338.54675 |
| Phobic | La.co02.04 | VII | 7 | 3 | 27.235 | 7.986 | 5.462 | | 11.931 | 23.357 | 105.108 | 476.40801 |
| Hydrophilic | La.co02.03 | IV | 0 | 1 | 26.186555 | | | | | | 19.460504 | 0 |
| Hydrophilic | La.co02.03 | IV | 1 | 1 | | | | | | | | |
| Hydrophilic | La.co02.03 | IV | 2 | 1 | | | | | | | | |
| Hydrophilic | La.co02.03 | IV | 3 | 1 | 15.449412 | 8.3213445 | 5.4742857 | 79.095052 | 5.8631933 | 2.7267227 | 17.815462 | -8.453234 |
| Hydrophilic | La.co02.03 | IV | 4 | 1 | 17.521345 | 8.610084 | 6.3203361 | | 5.0033613 | 5.9431933 | 21.529748 | 10.633043 |
| Hydrophilic | La.co02.03 | IV | 5 | 1 | 19.415798 | 6.472605 | 4.4369748 | 83.056287 | 6.1687395 | 23.234286 | 65.177479 | 234.92184 |
| Hydrophilic | La.co02.03 | IV | 6 | 1 | 9.3011765 | 5.3848739 | 3.9458824 | 84.931648 | 7.1684034 | 33.333445 | 98.162017 | 404.41662 |
| Hydrophilic | La.co02.03 | IV | 7 | 1 | 7.3236975 | 2.9781513 | 2.3781513 | 90.918426 | 7.245042 | 93.450756 | 212.4884 | 991.89567 |
| Hydrophilic | La.co02.03 | IV | 0 | 2 | 26.268571 | | | | | | 19.750252 | 0 |
| Hydrophilic | La.co02.03 | IV | 1 | 2 | | | | | | | | |
| Hydrophilic | La.co02.03 | IV | 2 | 2 | | | | | | | | |
| Hydrophilic | La.co02.03 | IV | 3 | 2 | 20.620168 | 7.6803361 | 7.1089076 | 72.937594 | 11.396975 | 11.130756 | 24.897815 | 26.063278 |
| Hydrophilic | La.co02.03 | IV | 4 | 2 | 16.823866 | 10.496471 | 10.496471 | 60.041715 | 13.310924 | 14.527731 | 31.198319 | 57.964157 |
| Hydrophilic | La.co02.03 | IV | 5 | 2 | 14.562017 | 9.1384874 | 5.947563 | 77.358635 | 11.640336 | 22.25042 | 67.821849 | 243.39738 |

Biology Open • Supplementary information

| | | | | | | | | | | | | |
|---|---|---|---|---|---|---|---|---|---|---|---|---|
| Hydrophilic | La.co02.03 | IV | 6 | 2 | 16.023193 | 6.7751261 | 5.2319328 | 80.082919 | 16.16605 | 39.129076 | 111.16471 | 462.85209 |
| Hydrophilic | La.co02.03 | IV | 7 | 2 | 8.5731092 | 2.2521008 | 2.2521008 | 91.426634 | 16.60437 | 117.38084 | 231.65143 | 1072.9037 |
| Hydrophilic | La.co02.03 | IV | 0 | 3 | 26.136471 | | | | | | 19.81479 | 0 |
| Hydrophilic | La.co02.03 | IV | 1 | 3 | | | | | | | | |
| Hydrophilic | La.co02.03 | IV | 2 | 3 | 24.092773 | 16.604034 | 10.843025 | 58.513812 | 12.545882 | 2.707563 | 20.099496 | 1.4368352 |
| Hydrophilic | La.co02.03 | IV | 3 | 3 | 29.512941 | 9.0887395 | 7.4406723 | 71.531457 | 9.4571429 | 7.9778151 | 33.903193 | 71.100443 |
| Hydrophilic | La.co02.03 | IV | 4 | 3 | 28.673277 | 8.4510924 | 5.9421849 | 77.264777 | 10.180504 | 13.73479 | 44.38521 | 124.00041 |
| Hydrophilic | La.co02.03 | IV | 5 | 3 | 24.350588 | 4.9630252 | 4.382521 | 83.232162 | 12.59395 | 43.746555 | 107.35294 | 441.78188 |
| Hydrophilic | La.co02.03 | IV | 6 | 3 | 16.990252 | 4.1966387 | 3.7146218 | 85.787592 | 26.893109 | 59.33042 | 155.08101 | 682.6528 |
| Hydrophilic | La.co02.03 | IV | 7 | 3 | 14.181513 | 5.3996639 | 3.2386555 | 87.608673 | 16.583529 | 101.86252 | 277.87126 | 1302.3427 |
| Hydrophilic | La.co02.03 | IV | 0 | 4 | 26.378487 | | | | | | 19.319664 | 0 |
| Hydrophilic | La.co02.03 | IV | 1 | 4 | | | | | | | | |
| Hydrophilic | La.co02.03 | IV | 2 | 4 | 19.206723 | 5.9331092 | 3.4278992 | | 7.3378151 | 10.488067 | 29.283697 | 51.57457 |
| Hydrophilic | La.co02.03 | IV | 3 | 4 | 21.512605 | 6.9092437 | 5.2127731 | | 12.334454 | 16.997647 | 50.598319 | 161.90062 |
| Hydrophilic | La.co02.03 | IV | 4 | 4 | 13.571765 | 6.777479 | 5.805042 | 77.993272 | 10.4 | 20.792269 | 62.492773 | 223.46719 |
| Hydrophilic | La.co02.03 | IV | 5 | 4 | 16.147227 | 4.9408403 | 4.7216807 | 82.10026 | 12.662521 | 59.164034 | 127.62924 | 560.61835 |
| Hydrophilic | La.co02.03 | IV | 6 | 4 | 13.133109 | 4.4497479 | 4.1381513 | 84.312401 | 14.84437 | 81.280672 | 169.78924 | 778.8416 |
| Hydrophilic | La.co02.03 | IV | 7 | 4 | | 4.1996639 | 4.1942857 | 84.099597 | 10.85042 | 113.02319 | 299.63294 | 1450.9221 |
| Phobic | La.co09.01 | VI | 0 | 1 | 24.88 | | | | | | 16.868 | 0 |
| Phobic | La.co09.01 | VI | 0 | 2 | 24.717 | | | | | | 17.337 | 0 |
| Phobic | La.co09.01 | VI | 1 | 2 | | | | | | | | |
| Phobic | La.co09.01 | VI | 2 | 2 | | | | | | | | |
| Phobic | La.co09.01 | VI | 3 | 2 | | | | | | | | |
| Phobic | La.co09.01 | VI | 4 | 2 | | | | | | | | |
| Phobic | La.co09.01 | VI | 5 | 2 | 10.682 | 10.949 | 7.183 | 70.93903 | 23.532 | 69.556 | 81.315 | 369.02578 |
| Phobic | La.co09.01 | VI | 6 | 2 | 8.492 | 8.194 | 4.398 | 82.206578 | 24.112 | 109.49 | 115.864 | 568.30478 |
| Phobic | La.co09.01 | VI | 7 | 2 | 6.998 | 11.414 | 5.689 | | 25.501 | 144.841 | 155.774 | 798.50609 |
| Phobic | La.co09.01 | VI | 0 | 3 | 24.771 | | | | | | 17.271 | 0 |
| Phobic | La.co09.01 | VI | 1 | 3 | | | | | | | | |
| Phobic | La.co09.01 | VI | 2 | 3 | | | | | | | | |
| Phobic | La.co09.01 | VI | 3 | 3 | | | | | | | | |

Biology Open • Supplementary information

| | | | | | | | | | | | | |
|---|---|---|---|---|---|---|---|---|---|---|---|---|
| Phobic | La.co09.01 | VI | 4 | 3 | | | | | | | | |
| Phobic | La.co09.01 | VI | 5 | 3 | 8.797 | 14.808 | 13.58 | 45.177829 | 46.499 | 38.807 | 84.291 | 388.04933 |
| Phobic | La.co09.01 | VI | 6 | 3 | 8.808 | 7.823 | 6.31 | 74.526664 | 35.557 | 50.635 | 119.139 | 589.82109 |
| Phobic | La.co09.01 | VI | 7 | 3 | 6.788 | 8.08 | 3.958 | 84.021638 | 31.485 | 41.131 | 206.971 | 1098.373 |
| Phobic | La.co09.01 | VI | 0 | 4 | 27.303 | | | | | | 19.596 | 0 |
| Phobic | La.co09.01 | VI | 1 | 4 | | | | | | | | |
| Phobic | La.co09.01 | VI | 2 | 4 | | | | | | | | |
| Phobic | La.co09.01 | VI | 3 | 4 | | | | | | | | |
| Phobic | La.co09.01 | VI | 4 | 4 | | | | | | | | |
| Phobic | La.co09.01 | VI | 5 | 4 | 30.412 | 10.348 | 9.265 | 66.066 | 26.229 | 48.104 | 134.991 | 588.87018 |
| Phobic | La.co09.01 | VI | 6 | 4 | 22.983 | 5.468 | 3.53 | 87.071018 | 27.407 | 61.008 | 158.706 | 709.88977 |
| Phobic | La.co09.01 | VI | 7 | 4 | 6.788 | 6.727 | 3.769 | | 14.007 | 42.94 | 224.307 | 1044.6571 |
| Phobic | La.co09.01 | VI | 0 | 5 | 25.027 | | | | | | 17.167 | 0 |
| Phobic | La.co09.01 | VI | 1 | 5 | | | | | | | | |
| Phobic | La.co09.01 | VI | 2 | 5 | 27.914 | 25.87 | 24.239 | 3.1485995 | 28.276 | 15.76 | 22.228 | 29.480981 |
| Phobic | La.co09.01 | VI | 3 | 5 | 42.174 | 13.794 | 12.245 | 51.072841 | 21.258 | 22.081 | 43.36 | 152.57762 |
| Phobic | La.co09.01 | VI | 4 | 5 | 44.79 | 16.343 | 14.753 | | 23.425 | 22.109 | 53.695 | 212.78033 |
| Phobic | La.co09.01 | VI | 5 | 5 | 14.384 | 6.668 | 6.445 | 74.247812 | 20.932 | 33.277 | 160.387 | 834.27506 |
| Phobic | La.co09.01 | VI | 6 | 5 | 5.21 | 5.356 | 4.279 | 82.902465 | 14.516 | 78.595 | 190.213 | 1008.0154 |
| Phobic | La.co09.01 | VI | 7 | 5 | 7.59 | 4.505 | 3.741 | 85.052144 | 13.903 | 106.983 | 260.442 | 1417.1084 |
| Phobic | La.co09.01 | VI | 0 | 6 | 25.941 | | | | | | 19.479 | 0 |
| Phobic | La.co09.01 | VI | 1 | 6 | | | | | | | | |
| Phobic | La.co09.01 | VI | 2 | 6 | 13.837 | 9.678 | 9 | 65.305886 | 14.492 | 28.459 | 55.987 | 187.42235 |
| Phobic | La.co09.01 | VI | 3 | 6 | 14.704 | 7.172 | 7.055 | 72.80367 | 18.85 | 13.539 | 83.535 | 328.84645 |
| Phobic | La.co09.01 | VI | 4 | 6 | 16.348 | 7.13 | 6.254 | 75.891446 | 16.398 | 17.554 | 97.462 | 400.34396 |
| Phobic | La.co09.01 | VI | 5 | 6 | 7.459 | 5.687 | 4.987 | 80.775606 | 22.917 | 72.585 | 191.578 | 883.51045 |
| Phobic | La.co09.01 | VI | 6 | 6 | 5.329 | 4.443 | 3.916 | 84.904206 | 20.493 | 93.835 | 226.313 | 1061.8307 |
| Phobic | La.co09.01 | VI | 7 | 6 | 7.661 | 4.338 | 4.259 | | 20.639 | 127.941 | 307.133 | 1476.7391 |
| Phobic | La.co09.01 | VI | 0 | 7 | 23.118 | | | | | | 16.745 | 0 |
| Phobic | La.co09.01 | VI | 1 | 7 | 14.007 | 10.154 | 8.452 | 63.439744 | 7.317 | 6.565 | 23.807 | 42.173783 |
| Phobic | La.co09.01 | VI | 2 | 7 | 12.192 | 7.392 | 5.993 | 74.076477 | 7.095 | 26.18 | 75.57 | 351.2989 |

| | | | | | | | | | | | | |
|---|---|---|---|---|---|---|---|---|---|---|---|---|
| Phobic | La.co09.01 | VI | 3 | 7 | | 5.852 | 4.448 | 80.759581 | 7.372 | 15.896 | 120.789 | 621.34368 |
| Phobic | La.co09.01 | VI | 4 | 7 | | 5.077 | 4.677 | | 5.982 | 7.033 | 130.143 | 677.20514 |
| Phobic | La.co09.01 | VI | 5 | 7 | | 4.882 | 3.481 | 84.942469 | 6.614 | 8.198 | 291.502 | 1640.8301 |
| Phobic | La.co09.01 | VI | 6 | 7 | | 3.531 | 3.025 | 86.914958 | 7.06 | 11.883 | 329.166 | 1865.7569 |
| Phobic | La.co09.01 | VI | 7 | 7 | | | | | | | | |
| Hydrophilic | La.co09.02 | V | 0 | 1 | 20.542 | | | | | | 14.583866 | 0 |
| Hydrophilic | La.co09.02 | V | 1 | 1 | | | | | | | | |
| Hydrophilic | La.co09.02 | V | 2 | 1 | | | | | | | | |
| Hydrophilic | La.co09.02 | V | 3 | 1 | 11.426 | 8.021 | 7.168 | 65.105637 | 14.935 | 25.079 | 30.414 | 108.54553 |
| Hydrophilic | La.co09.02 | V | 4 | 1 | 10.309 | 9.285 | 7.904 | | 14.063 | 28.536 | 35.966 | 146.615 |
| Hydrophilic | La.co09.02 | V | 5 | 1 | 7.219 | 8.77 | 7.074 | 65.563236 | 14.558 | 22.473 | 39.741 | 172.49977 |
| Hydrophilic | La.co09.02 | V | 6 | 1 | 10.053 | 8.255 | 5.827 | 71.633726 | 17.518 | 43.91 | 58.509 | 301.18993 |
| Hydrophilic | La.co09.02 | V | 7 | 1 | | 6.348 | 3.982 | 80.615325 | 11.614 | 94.002 | 109.252 | 649.12923 |
| Hydrophilic | La.co09.02 | V | 0 | 2 | 20.896 | | | | | | 13.809076 | 0 |
| Hydrophilic | La.co09.02 | V | 1 | 2 | | | | | | | | |
| Hydrophilic | La.co09.02 | V | 2 | 2 | | | | | | | | |
| Hydrophilic | La.co09.02 | V | 3 | 2 | | | | | | | 30.822 | 123.20104 |
| Hydrophilic | La.co09.02 | V | 4 | 2 | | | | | | | 46.425 | 236.19195 |
| Hydrophilic | La.co09.02 | V | 5 | 2 | | | | | | | 49.654 | 259.57512 |
| Hydrophilic | La.co09.02 | V | 6 | 2 | | | | | | | 57.551 | 316.76215 |
| Hydrophilic | La.co09.02 | V | 7 | 2 | | | | | | | 116.642 | 744.67638 |
| Hydrophilic | La.co09.02 | V | 0 | 3 | 19.983 | | | | | | 14.29479 | 0 |
| Hydrophilic | La.co09.02 | V | 1 | 3 | | | | | | | | |
| Hydrophilic | La.co09.02 | V | 2 | 3 | 14.147 | 10.149 | 9.926 | 50.327779 | | 13.481 | 19.568 | 36.889035 |
| Hydrophilic | La.co09.02 | V | 3 | 3 | 6.116 | 7.296 | 6.166 | 69.143772 | 12.639 | 41.782 | 41.782 | 192.28831 |
| Hydrophilic | La.co09.02 | V | 4 | 3 | 5.718 | 5.577 | 5.151 | 74.22309 | 14.844 | 35.562 | 50.782 | 255.24831 |
| Hydrophilic | La.co09.02 | V | 5 | 3 | 5.724 | 6.705 | 4.746 | 76.249812 | 14.383 | 34.575 | 52.87 | 269.85503 |
| Hydrophilic | La.co09.02 | V | 6 | 3 | 5.252 | 5.551 | 4.67 | 76.630136 | 15.551 | 70.151 | 76.645 | 436.17437 |
| Hydrophilic | La.co09.02 | V | 7 | 3 | 7.743 | 7.857 | 4.151 | 79.227343 | 20.341 | 65.89 | 155.951 | 990.96392 |
| Hydrophilic | La.co09.02 | V | 0 | 4 | 19.187 | | | | | | 15.107227 | 0 |
| Hydrophilic | La.co09.02 | V | 1 | 4 | 10.413 | 6.718 | 6.718 | 64.98671 | 8.832 | 13.793 | 20.169 | 33.50564 |

Biology Open • Supplementary information

| | | | | | | | | | | | | |
|---|---|---|---|---|---|---|---|---|---|---|---|---|
| Hydrophilic | La.co09.02 | V | 2 | 4 | 11.507 | 7.933 | 7.037 | | 10.424 | 17.669 | 33.221 | 119.90138 |
| Hydrophilic | La.co09.02 | V | 3 | 4 | 8.298 | 7.427 | 5.656 | 70.521707 | 15.041 | 57.937 | 66.298 | 338.84957 |
| Hydrophilic | La.co09.02 | V | 4 | 4 | 8.825 | 5.958 | 4.699 | 75.50946 | 15.134 | 74.871 | 78.548 | 419.93659 |
| Hydrophilic | La.co09.02 | V | 5 | 4 | 4.819 | 6.248 | 2.821 | | 17.507 | 77.946 | 81.84 | 441.72748 |
| Hydrophilic | La.co09.02 | V | 6 | 4 | 4.294 | 5.491 | 3.609 | 81.190389 | 14.295 | 93.124 | 102.429 | 578.01325 |
| Hydrophilic | La.co09.02 | V | 7 | 4 | 11.586 | 3.95 | 3.142 | 83.624329 | 15.829 | 59.478 | 192.129 | 1171.7688 |
| Hydrophilic | La.co09.02 | V | 0 | 5 | 20.125 | | | | | | 15.134454 | 0 |
| Hydrophilic | La.co09.02 | V | 1 | 5 | 10.9 | 4.51 | 4.51 | 77.590062 | 17.14 | 16.072 | 35.835 | 136.77762 |
| Hydrophilic | La.co09.02 | V | 2 | 5 | 5.053 | 6.134 | 3.336 | | 17.185 | 28.681 | 40.406 | 166.98023 |
| Hydrophilic | La.co09.02 | V | 3 | 5 | 5.383 | 7.18 | 5.077 | 74.772671 | 22.575 | 81.606 | 89.032 | 488.27363 |
| Hydrophilic | La.co09.02 | V | 4 | 5 | 5.221 | 6.272 | 3.742 | 81.406211 | 19.392 | 87.534 | 92.887 | 513.74531 |
| Hydrophilic | La.co09.02 | V | 5 | 5 | 5.383 | 6.527 | 3.549 | 82.365217 | 19.383 | 89.092 | 96.874 | 540.08917 |
| Hydrophilic | La.co09.02 | V | 6 | 5 | 6.339 | 5.43 | 4.376 | | 19.491 | 100.929 | 122.628 | 710.25719 |
| Hydrophilic | La.co09.02 | V | 7 | 5 | 11.219 | 4.003 | 1.707 | 91.518012 | 15.414 | 35.85 | 216.905 | 1333.1868 |

Biology Open • Supplementary information
